# Targeting ATAD3A Phosphorylation Mediated by TBK1 Ameliorates Senescence‐Associated Pathologies

**DOI:** 10.1002/advs.202404109

**Published:** 2024-11-08

**Authors:** Yujiao He, Yanchen Liu, Mingyue Zheng, Yuxiu Zou, Mujie Huang, Linsheng Wang, Ge Gao, Zhongjun Zhou, Guoxiang Jin

**Affiliations:** ^1^ Guangdong Cardiovascular Institute Medical Research Institute Guangdong Provincial People's Hospital (Guangdong Academy of Medical Sciences) Southern Medical University Guangzhou 510080 China; ^2^ School of Biomedical Sciences The University of Hong Kong Hong Kong China; ^3^ Orthopedic Center University of Hong Kong‐Shenzhen Hospital No.1, Haiyuan 1st Road, Futian Shenzhen 518053 China; ^4^ Guangdong Provincial Geriatrics Institute Guangdong Provincial People's Hospital Guangdong Academy of Medical Sciences Guangzhou 510080 China

**Keywords:** anti‐aging therapy, cellular senescence, chemotherapy, mitophagy, TBK1‐ATAD3A axis

## Abstract

Targeting cellular senescence, one of the hallmarks of aging and aging‐related pathologies emerges as an effective strategy for anti‐aging and cancer chemotherapy. Here, a switch from TBK1‐OPTN axis to TBK1‐ATAD3A axis to promote cellular senescence is shown. Mechanically, TBK1 protein is abnormally activated and localized to the mitochondria during senescence, which directly phosphorylates ATAD3A at Ser321. Phosphorylated ATAD3A is significantly elevated in cellular senescence as well as in physiological and pathological aging and is essential for suppressing Pink1‐mediated mitophagy by facilitating Pink1 mitochondrial import. Inhibition of ATAD3A phosphorylation at Ser321 by either TBK1 deficiency or by a Ser321A mutation rescues the cellular senescence. A blocking peptide, TAT‐PEP, specifically abrogating ATAD3A phosphorylation, results in elevated cell death by preventing doxorubicin‐induced senescence, thus leading to enhanced tumor sensitivity to chemotherapy. TAT‐PEP treatment also ameliorates various phenotypes associated with physiological aging. Collectively, these results reveal the TBK1‐ATAD3A‐Pink1 axis as a driving force in cellular senescence and suggest a potential mitochondrial target for anti‐aging therapy.

## Graphical abstract

This study introduces a novel TBK1‐ATAD3A axis in cellular senescence, highlighting a strategic target for aging and cancer treatments. It is demonstrated that TBK1 activates and localizes to mitochondria, phosphorylating ATAD3A and thereby accelerating cellular senescence by inhibiting mitophagy. Targeting ATAD3A phosphorylation with the peptide TAT‐PEP shows exceptional anti‐aging and anti‐tumor effects, providing a potential therapeutic strategy for combating aging and aging‐associated disorders.

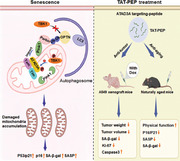

## 1 Introduction

Cellular senescence, defined by a state of permanent cell cycle arrest, can be triggered by various inducers such as telomere dysfunction, DNA damage, and oxidative stress.^[^ ^1^, ^2^ ^]^ Despite exit from proliferation, senescent cells remain metabolically active with enlarged and flattened morphology as well as upregulated biomarkers indicative of cellular senescence, including senescence‐associated‐β‐galactosidase (SA‐β‐gal), p16, p21, and p53.^[^ ^3^, ^4^, ^5^, ^6^, ^7^ ^]^ Additionally, senescent cells release a wide range of factors, such as inflammatory cytokines, chemokines, and growth factors, termed as the senescence‐associated secretory phenotype (SASP).^[^ ^1^, ^8^ ^]^ These factors can have multiple effects on the surrounding tissues and contribute to the development of age‐related degenerative diseases and tumorigenesis.^[^ ^9^, ^10^ ^]^ Interventions that target cellular senescence by either eliminating or preventing senescent cells have been demonstrated to extend lifespan and alleviate tissue damage in multiple animal models.^[^ ^11^, ^12^, ^13^ ^]^ Thus, characterizing specific senescence markers or modulators is beneficial for identifying senescent cells and developing druggable targets.

TBK1 (TANK‐binding kinase 1) is a multifunctional serine/threonine protein kinase involved in a variety of cellular processes, including innate immune, inflammatory cytokine production, cell growth, and autophagy.^[^ ^14^ ^]^ The abnormal activation of TBK1 is often associated with the development of various diseases, such as certain types of cancer, autoimmune diseases, and neurodegenerative disorders.^[^ ^15^ ^]^ Evidence shows that TBK1 contributes to tau hyperphosphorylation and neuronal loss in human Alzheimer's disease (AD) and parkinsonism, suggesting its crucial role in maintaining neural health during aging.^[^ ^16^, ^17^ ^]^ However, TBK1's role in aging and cellular senescence remains largely unexplored and warrants further investigation. Additionally, the function of TBK1 is closely related to its subcellular localization, which is determined by its selective interaction with specific adaptor proteins in response to different upstream stimuli. For example, TBK1 binds to TANK at the perinuclear region to induce the production of IFN during viral infections.^[^ ^18^ ^]^ Conversely, TBK1 associates with OPTN in autophagosomes, playing a role in mitophagy under conditions of cellular stress.^[^ ^19^ ^]^ Thus, focusing on the localization of TBK1 may provide insights into its unique substrates and functions.

The ATPase family AAA domain‐containing 3A (ATAD3A), a mitochondrial protein that regulates nucleoid organization, and cholesterol metabolism, serves as a scaffold to maintain the integrity of mitochondrial membrane structure.^[^ ^20^, ^21^, ^22^, ^23^ ^]^ Our previous findings demonstrate that ATAD3A acts as a suppressor of Pink1‐mediated mitophagy and plays a key role in maintaining hematopoietic homeostasis and tumor chemoimmunotherapy resistance.^[^ ^24^, ^25^ ^]^ However, the role of ATAD3A in the aging process is poorly understood. Recent findings reveal that ATAD3A is the key protein behind Huntington's disease and Alzheimer's disease,^[^ ^26^, ^27^ ^]^ indicative of a potential link between ATAD3A and aging.

Mitophagy acts as a protective mechanism to selectively remove dysfunctional or redundant mitochondria and plays an essential role in mitochondrial homeostasis, as well as in cell death and metabolism.^[^ ^28^ ^]^ Growing evidence indicates that mitophagy declines with age, which could contribute to the accumulation of damaged mitochondria during aging, and is associated with various age‐related diseases, such as degenerative disorders, cardiovascular diseases, and cancer.^[^ ^29^, ^30^, ^31^, ^32^, ^33^ ^]^ Hence, understanding the regulatory mechanisms of mitophagy will provide insights into the development of therapeutic strategies against aging and age‐associated disorders. PINK1/Parkin‐mediated pathway is the best‐known mitophagy pathway among many others. This pathway functions to remove damaged mitochondria in a ubiquitination‐dependent manner. Pink1 accumulates on the surface of damaged mitochondria and recruits Parkin to ubiquitinate downstream mitochondrial proteins; the ubiquitinated proteins then recruit LC3 through mitophagy adaptors to initiate mitophagy.^[^ ^34^ ^]^ In healthy mitochondria, Pink1 is imported by two mitochondrial membrane complexes, TOM and TIM, for processing and degradation, thereby escaping mitophagy.^[^ ^34^ ^]^ Loss of Pink1 in mice causes numerous age‐related disorders, such as neuronal and muscular degeneration and cardiomyopathies.^[^ ^35^, ^36^, ^37^, ^38^ ^]^ Decreased Pink1 has been observed in multiple aging‐related diseases such as heart failure, idiopathic pulmonary fibrosis (IPF), and Alzheimer's disease.^[^ ^39^, ^40^, ^41^ ^]^ Theses imply a crucial role for Pink1‐mediated mitophagy in aging. However, the mechanism driving the deficiency of Pink1 during aging remains largely unclear.

In this study, we identified an increased activation and mitochondrial localization of TBK1 in senescent cells, along with a shift of TBK1‐OPTN axis to TBK1‐ATAD3A axis. TBK1 phosphorylated ATAD3A at Ser321 site to promote cellular senescence. Significantly increased ATAD3A phosphorylation was detected by an antibody specific for Ser321 phosphorylation in various senescent cell models and in mouse tissues from physiological aging as well as pathological aging. To test if ATAD3A Ser321 phosphorylation may serve as a druggable target for the intervention of aging and aging‐associated pathologies, a blocking peptide TAT‐PEP which specifically abrogates the phosphorylation of ATAD3A was examined. Notably, TAT‐PEP prevented doxorubicin‐induced cellular senescence, switched the cells to death, and sensitized tumor cells to chemotherapy both in vitro and in vivo. In addition, TAT‐PEP improved aging‐related pathological phenotypes in physiologically aged mice. Lastly, we showed that the phosphorylation of ATAD3A mediated by TBK1 is required for ATAD3A‐facilitated cellular senescence by inhibiting Pink1‐dependent mitophagy, revealing that the TBK1‐ATAD3A‐Pink1 axis is a key regulator between cellular senescence and mitophagy. Taken together, our study demonstrates the pivotal role of the TBK1‐ATAD3A‐Pink1 axis in promoting cellular senescence and provides a potential therapeutic strategy by targeting ATAD3A phosphorylation for the intervention of aging and aging‐associated disorders.

## 2 Results

### 2.1 TBK1 is Abnormally Activated and localizes to Mitochondria in Senescent Cells

**Figure 1:**
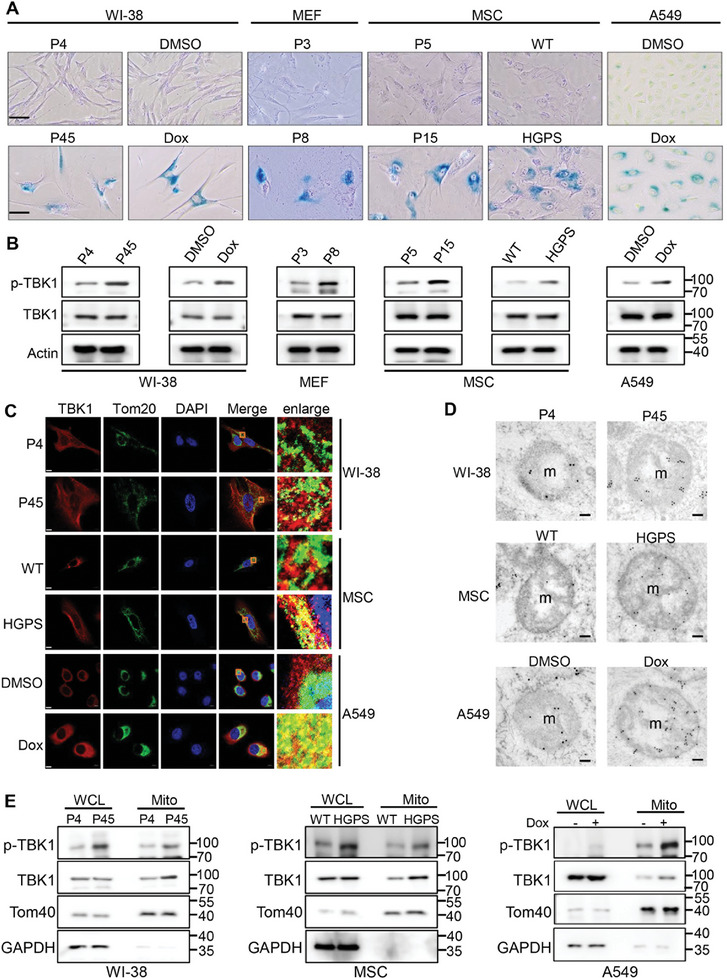
Increased activation and mitochondrial localization of TBK1 in senescent cells. A) SA‐β‐gal staining of normal and senescent cells in various senescent cell models (*n =* 3). P: passages, Dox: Doxorubicin (150 nm), WT: wild type, HGPS: Hutchinson–Gilford progeria syndrome. Scale bars, 50 µm. B) Immunoblot analysis of phosphorylated TBK1 (pS172‐TBK1) and TBK1 in normal or senescent cells (*n =* 3). C) Confocal imaging of TBK1 (red) and Tom20 (green) co‐localization in normal or senescent cells (*n =* 3). Areas outlined by squares in the merged images are enlarged at right. Scale bars, 5 µm. D) Transmission electron microscopy (TEM) images of TBK1 labeled by antibody‐conjugated gold particles in normal or senescent cells (*n =* 3). m: mitochondria. Scale bars, 100 nm. E) Immunoblot analysis of p‐TBK1 and TBK1 in the mitochondrial fraction of normal or senescent cells (*n =* 3). Tom40: mitochondria marker, GAPDH: whole cell marker.

To investigate the potential role of TBK1 in cellular senescence, we established multiple senescent cell models using WI‐38, MEF, MSC, and A549 cell lines. Senescence was induced by various methods, including long‐term culture and treatment with doxorubicin (a chemotherapeutic agent known to induce cellular senescence). Additionally, fibroblasts derived from a patient with Hutchinson–Gilford Progeria Syndrome (HGPS) were used to generate MSCs as a cell model of premature senescence.^[^ ^42^ ^]^ The senescence states were verified by cellular senescence markers, including higher SA‐β‐gal activity, increased expression of p53, p21, p16, and reduced proliferation (**Figure** **1**A; Figure S1A,B, Supporting Information). Activation of TBK1 is triggered by the trans‐autophosphorylation at serine 172 (S172) located in the activation loop of the kinase domain. Therefore, we measured activated TBK1 levels by using a pS172‐TBK1 antibody. We detected an increase in pS172‐TBK1 levels in the senescent cell models (Figure 1B; Figure S1C, Supporting Information). Furthermore, both confocal microscopy and immunoelectron microscopy observations showed a significant increase in TBK1 within the mitochondria of senescent cells compared to normal cells (Figure 1C,D). By biochemically isolating mitochondria, we observed higher levels of activated TBK1 in the mitochondria of senescent cells compared to normal cells (Figure 1E; Figure S1D, Supporting Information). Using a mitochondrial protease protection assay, we found that TBK1 is primarily localized in the mitochondrial matrix (Figure S1E, Supporting Information). These findings indicate that activated TBK1 is intricately linked to the process of cellular senescence, potentially exerting its effect through interactions with mitochondrial proteins.

### 2.2 TBK1 Phosphorylates ATAD3A to Accelerate Senescence

**Figure 2:**
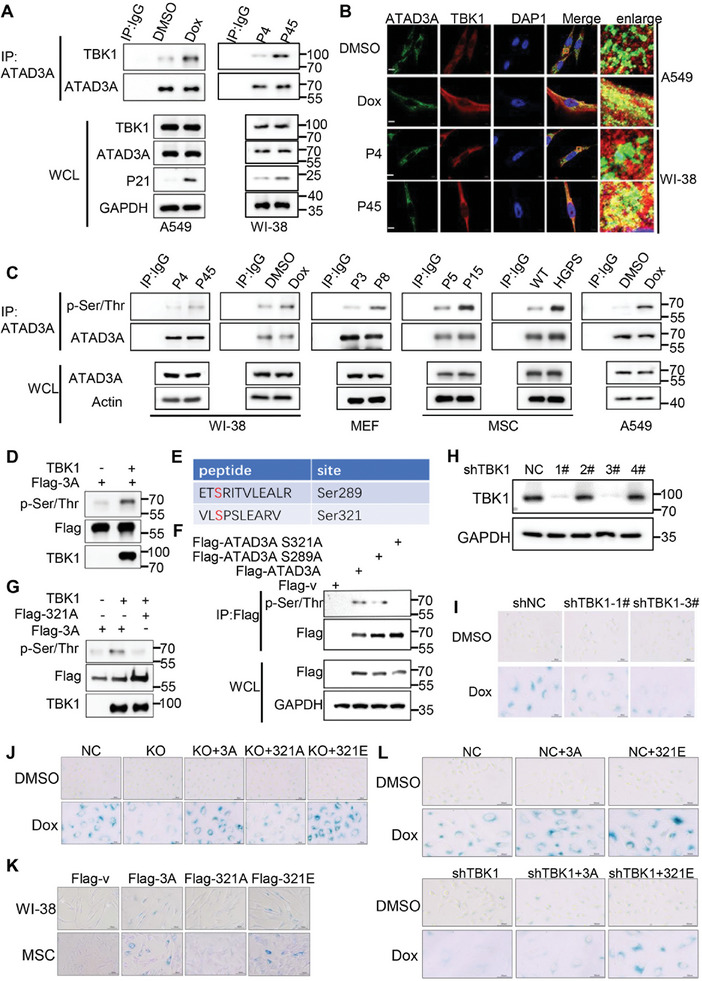
TBK1 phosphorylates ATAD3A at Ser321 to promote cellular senescence. A) Immunoblot analysis of the interaction between endogenous ATAD3A and TBK1 in normal or senescent A549 and WI‐38 cells (*n =* 3). B) Confocal imaging of ATAD3A (green) and TBK1 (red) co‐localization in normal or senescent A549 and WI‐38 cells (*n =* 3). Areas outlined by squares in the merged images are enlarged at right. Scale bars, 5 µm. C) Immunoblot analysis of phosphorylated ATAD3A in senescent cells using a p‐Ser‐ and p‐Thr‐specific antibody (*n =* 3). D) In vitro TBK1 kinase assay using recombinant TBK1 and Flag‐ATAD3A purified from 293T cells as the substrate (*n =* 3). E) Listing of 2 ATAD3A Ser phosphorylation sites (highlighted in red) identified via MS analysis (*n =* 2). F) Immunoblot analyzing the phosphorylation of transfected Flag‐ATAD3A or Ser‐mutated Flag‐ATAD3A (*n =* 3). G) In vitro TBK1 kinase assay using recombinant TBK1 and Flag‐ATAD3A, Flag‐ATAD3A S321A purified from stable A549 cell lines as the substrate (*n =* 3). H) Immunoblot analysis of TBK1 in stable negative control (shNC) or shTBK1‐expressing A549 cells (*n =* 3). I) SA‐β‐gal staining of shNC and shTBK1 A549 cells. Cells were initially induced with doxorubicin (150 nm) for 48 h, followed by additional culturing for 48 h. Scale bars, 100 µm. J) SA‐β‐gal staining of NC and ATAD3A KO A549 cells stably expressing Flag‐ATAD3A, Flag‐ATAD3A S321A, or Flag‐ATAD3A S321E (*n =* 3). Cells were initially induced with doxorubicin (150 nm) for 48 h, followed by additional culturing for 48 h. Scale bars, 100 µm. K) SA‐β‐gal staining of WI‐38 and MSC cells transfected with Flag‐ATAD3A, Flag‐ATAD3A S321A or Flag‐ATAD3A S321E (*n =* 3). Scale bars, 100 µm. L) SA‐β‐gal staining of shNC, shTBK1, and shNC or shTBK1 plus simultaneously expressed Flag‐ATAD3A or Flag‐ATAD3A S321E A549 cells (*n =* 3). Cells were initially induced with doxorubicin (150 nm) for 48 h, followed by additional culturing for 48 h. Scale bars, 100 µm.

We next sought to explore the potential interactions of TBK1 with mitochondrial proteins by a mass spectrometry assay. We identified that the mitochondrial protein ATAD3A could interact with TBK1 (Figure S2A, Supporting Information). Supporting this finding, ATAD3A was found to interact with and colocalize with TBK1 (**Figure** **2**A,B; Figure S2B, Supporting Information). Moreover, this interaction and colocalization were enhanced in senescent cells compared to normal cells (Figure 2A,B; Figure S2B, Supporting Information). Given that TBK1 is a serine/threonine protein kinase, we speculated that TBK1 might phosphorylate ATAD3A. Consistent with our speculation, phosphorylation of ATAD3A was significantly increased in the senescent cell models, while neither mRNA levels nor protein levels of ATAD3A changed in the senescent cell models (Figure 2C; Figure S2C, Supporting Information). In vitro kinase assay confirmed that TBK1 directly phosphorylated ATAD3A (Figure 2D). We further identified two potential serine phosphorylation sites on ATAD3A by mass spectrometry analysis (Figure 2E). By individually mutating each of these residues to alanine, we confirmed that the mutation of Ser321, but not Ser289, reduced the phosphorylation of ATAD3A (Figure 2F). Additionally, we found that TBK1 phosphorylated Flag‐ATAD3A but not Flag‐ATAD3A S321A, confirming TBK1 as the upstream kinase for ATAD3A Ser321 phosphorylation (Figure 2G).

To investigate the roles of TBK1 phosphorylating ATAD3A in cellular senescence, we first generated stable TBK1 knockdown A549 cell lines using short hairpin RNAs (shRNAs) (Figure 2H). The reduction of TBK1 markedly decreased SA‐β‐gal activity and the expression of p53, and p21 after senescence induction (Figure 2I; Figure S2D,E, Supporting Information). Next, we generated ATAD3A knockout (KO) A549 cell lines (Figure S2F, Supporting Information), and simultaneously restored the cells with wild‐type (WT, ATAD3A), phosphor‐dead (S321A, ATAD3A S321A), or phosphor‐mimicking (S321E, ATAD3A S321E) mutants. ATAD3A KO suppressed doxorubicin‐induced cellular senescence, as evidenced by a remarkable reduction in SA‐β‐gal activity and lower expression of p53, p21, and p16 (Figure 2J; Figure S2G,H, Supporting Information). The inhibition was reversed by restoration with ATAD3A WT or ATAD3A S321E but not with ATAD3A S321A (Figure 2J; Figure S2G,H, Supporting Information). We also overexpressed ATAD3A WT and the related mutants in WI‐38 and MSC cell lines. Consistently, ATAD3A overexpression driven cellular senescence, which was prevented by ATAD3A S321A but further enhanced by ATAD3A S321E (Figure 2K; Figure S2I–K, Supporting Information). We reintroduced ATAD3A WT and ATAD3A S321E into the TBK1 knockdown cells. ATAD3A S321E significantly increased cellular senescence, but ATAD3A WT was not able to reverse senescence suppression in TBK1 deficient cells (Figure 2L; Figure S2L,M, Supporting Information), suggesting that ATAD3A phosphorylation mediates TBK1‐promoting cellular senescence. Collectively, our data demonstrate that TBK1 is the upstream kinase of ATAD3A and accelerates cellular senescence by mediating Ser321 phosphorylation.

### 2.3 ATAD3A Ser321 Phosphorylation is a Potential Marker of Cellular Senescence and Aging

**Figure 3:**
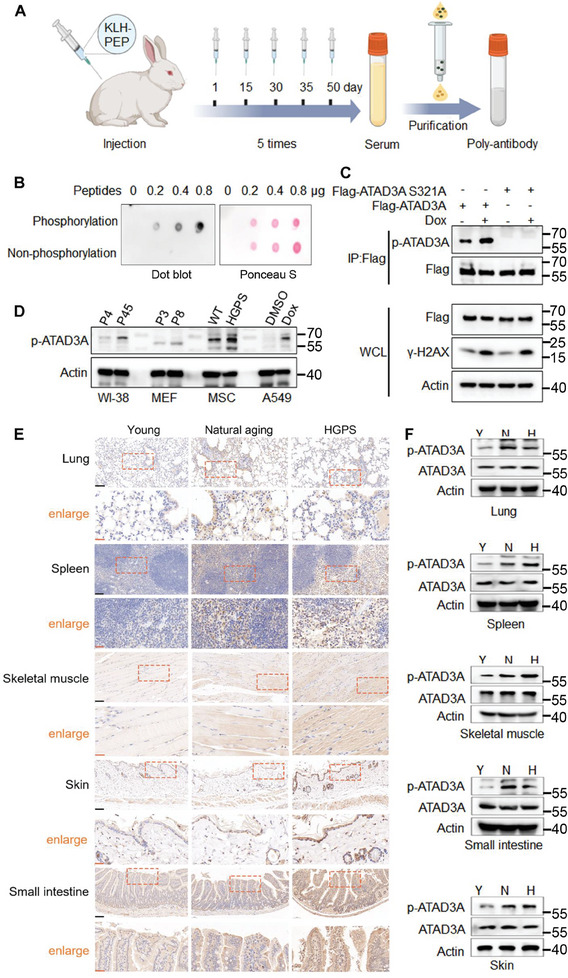
Enhanced ATAD3A Ser321 phosphorylation in naturally aged and prematurely aged mice. A) Diagram of antibody preparation. The peptide (LS(p)PSLEARVRDIA‐C) conjugated with KLH was used to immunize New Zealand White rabbits. After 5 immunizations, collected serum and the generated antibody were purified through affinity purification. B) Ser321 phosphorylated and non‐phosphorylated peptides (0.2–0.8 µg) of ATAD3A were dissolved in ddH_2_O, added onto the PVDF membranes, and followed by immunoblot analysis using Ser321 phosphorylation‐specific antibody (anti‐p‐ATAD3A) (*n =* 3). Ponceau S staining was used to verify the presence of peptides. C) Immunoblot analysis of Ser321 phosphorylation of stable expressing Flag‐ATAD3A WT and Flag‐ATAD3A S321A in A549 cells with or without doxorubicin (150 nm) treatment using p‐ATAD3A antibody (*n =* 3). D) Immunoblot analysis of ATAD3A phosphorylation in multiple normal and senescent cells using p‐ATAD3A antibody. E) Immunohistochemistry staining of p‐ATAD3A positive cells in the lung, spleen, skeletal muscle, skin, and small intestine from young (3 months), natural aging (20‐21 months) and HGPS mice (6 months) (*n =* 3). Areas outlined by squares are enlarged below. Scale bars (black), 60 µm. Scale bars (orange), 20 µm. F) Immunoblot analysis of p‐ATAD3A and ATAD3A in the lung, spleen, skeletal muscle, skin, and small intestine from young (Y), natural aging (N), and HGPS (H) mice (*n =* 3).

Sequence alignment of ATAD3A showed that the Ser321 sites are conserved across various species (Figure S3A, Supporting Information). We generated a Ser321 phosphorylation‐specific antibody (anti‐p‐ATAD3A) (**Figure** **3**A), which recognized the ATAD3A peptide containing phosphorylated Ser321, but not the non‐phosphorylated peptide, in a dot‐blot assay (Figure 3B). Additionally, the p‐ATAD3A antibody recognized phosphorylated ATAD3A WT, while the phosphorylation of ATAD3A S321A was barely observed (Figure 3C; Figure S3B, Supporting Information), indicating the high specificity of this antibody for detecting Ser321 phosphorylation of ATAD3A. Using the specific antibody, we verified that ATAD3A Ser321 phosphorylation was significantly increased in various senescent cell models (Figure 3D; Figure S3C, Supporting Information).

In order to explore the physiological significance of Ser321 phosphorylation in vivo, we performed immunohistochemistry (IHC) on various tissues of young, naturally aged, and prematurely aged (HGPS) mice. The tissues including the lung, spleen, skeletal muscle, skin, small intestine, and brown adipose, all showed higher ATAD3A Ser321 phosphorylation in naturally aged and HGPS mice compared to young mice (Figure 3E; Figure S3D, Supporting Information). Immunoblot analysis verified the increased ATAD3A phosphorylation in both naturally aged and HGPS mice (Figure 3F; Figure S3E, Supporting Information). These data suggested that phosphorylated ATAD3A may be an important marker indicative of aging. It is worth mentioning that in the heart, kidney, and liver tissues of both naturally aged and HGPS mice, phosphorylated ATAD3A showed slight or no significant changes (Figure S3D, Supporting Information), implying that ATAD3A phosphorylation exhibits tissue‐specific patterns. Collectively, our findings suggest that ATAD3A Ser321 phosphorylation may act as a new potential marker of cellular senescence and aging.

### 2.4 ATAD3A Phosphorylation Blocking Peptide TAT‐PEP Prevents Senescence and Sensitizes Tumor to Chemotherapy

**Figure 4:**
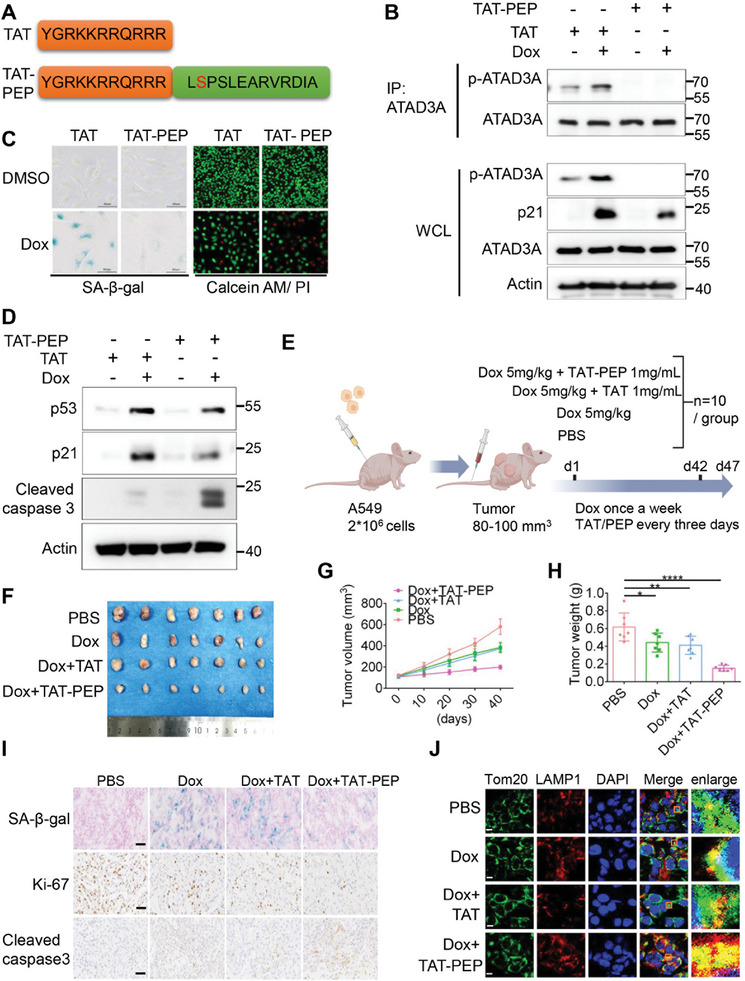
Blocking phosphorylation of ATAD3A via specific peptides increases tumor sensitivity to doxorubicin. A) Amino acid sequences of control peptide TAT and ATAD3A phosphorylation blocking peptide TAT‐PEP. B) Immunoblot analysis of ATAD3A phosphorylation in A549 cells treated with DMSO or doxorubicin (150 nm) in combination with TAT or TAT‐PEP. Cells were initially induced with DMSO or doxorubicin (150 nm) in combination with TAT (50 µm) or TAT‐PEP (50 µm) for 48 h, followed by additional culturing for 48 h (*n =* 3). C) Detection of cellular senescence and cell death in A549 cells treated with doxorubicin (150 nm) plus TAT (50 µm) or TAT‐PEP (50 µm), using SA‐β‐gal staining and Calcein AM/PI staining (*n =* 3). Scale bars, 100 µm. D) Immunoblot analysis of cellular senescence marker (p53, p21) and cell apoptosis marker (cleaved caspase 3) in A549 cells treated with doxorubicin (150 nm) plus TAT or TAT‐PEP (50 µM) (*n =* 3). E) Schematic diagram of the experimental design: 2×10^6^ A549 cells were subcutaneous (SC) injected into nude/BALB/C mice. When tumors reached 80–100 mm^3^, mice were treated via tail vein injection with doxorubicin (5 mg kg^−1^) plus TAT (100 µL, 1 mg mL^−1^) or TAT‐PEP (100 µL, 1 mg mL^−1^). PBS was used as a control. Doxorubicin injections were administered once weekly for six weeks, while PBS/TAT/TAT‐PEP injections were given every three days for the same duration. Experiments were conducted 5 days after the last injection. F) Representative images of tumors (*n =* 7 for each group, respectively). G) Tumor growth curves for mice treated with PBS, doxorubicin, doxorubicin plus TAT, and doxorubicin plus TAT‐PEP (*n =* 7 for each group, respectively). H) Tumor weights of mice after treatment with PBS, doxorubicin, doxorubicin plus TAT, and doxorubicin plus TAT‐PEP (*n =* 7 for each group, respectively). *p < 0.05, **p < 0.01, ****p < 0.0001 (one‐way ANOVA with Dunnet post‐hoc test). I) SA‐β‐gal staining and immunohistochemistry staining of senescent cells and Ki‐67, cleaved caspase 3 positive cells in mouse tumors (*n =* 3). Scale bars, 10 µm. J) Confocal imaging of Tom20 (green) and LAMP1 (red) in mouse tumors (*n =* 3). Areas outlined by squares in the merged images are enlarged at right. Scale bars, 5 µm. All data are presented as the mean ± SD.

Doxorubicin, a widely used chemotherapeutic agent, is known for its efficacy in inducing cell death (e.g., apoptosis) in rapidly dividing cancer cells. However, it can also induce cellular senescence in tumor cells, emerging as a potential driving force of chemotherapy resistance and tumor progression.^[^ ^43^, ^44^ ^]^ Given that TBK1‐mediated ATAD3A Ser321 phosphorylation is necessary for doxorubicin‐induced cellular senescence, we investigated whether targeting ATAD3A phosphorylation could be therapeutically beneficial. To block ATAD3A phosphorylation, we synthesized a competitive peptide containing non‐phosphorylated ATAD3A Ser321 site (320–332 aa) with a trans‐activator of transcription (TAT) tag placed in its N‐terminal region (TAT‐PEP), and a TAT domain as a negative control (TAT) (**Figure** **4**A). To determine whether the TAT‐PEP can inhibit ATAD3A phosphorylation, we treated A549 cells with TAT‐PEP in combination with doxorubicin. We found that TAT‐PEP significantly reduced ATAD3A Ser321 phosphorylation despite doxorubicin treatment (Figure 4B; Figure S4A, Supporting Information). Additionally, we conducted an assay to assess the potential off‐target effects of TAT‐PEP using a specific antibody against phosphorylated Serine and Threonine. No significant reduction in general phosphorylation levels was observed (Figure S4B, Supporting Information), suggesting that the inhibitory action of TAT‐PEP is highly specific to ATAD3A phosphorylation. Furthermore, TAT‐PEP markedly inhibited doxorubicin‐induced cellular senescence, as indicated by lower SA‐β‐gal activity and decreased expression of p53 and p21 compared to the TAT control (Figure 4C,D; Figure S4C, Supporting Information). Importantly, TAT‐PEP sensitized A549 cells to doxorubicin treatment, resulting in more cells undergoing death compared to the TAT control (Figure 4C). Notably, the increased cell death was accompanied by the elevated level of the apoptosis marker cleaved caspase 3 (Figure 4D), further indicating that TAT‐PEP enhances doxorubicin‐induced apoptosis. Similar results were also observed in WI‐38 cells (Figure S4D,E, Supporting Information).

We next investigated whether TAT‐PEP can promote the efficacy of doxorubicin treatment by using an A549 lung adenocarcinoma tumor model established in nude (nude/BALB/c) mice. When the tumors reached ≈80–100 mm^3^, we administered TAT‐PEP or TAT control peptides in combination with doxorubicin to the mice (Figure 4E). All mice were sacrificed 47 days later. Despite that doxorubicin alone or in combination with TAT significantly inhibited tumor growth compared to the negative control (PBS) (Figure 4F–H), the tumors in the group treated with doxorubicin plus TAT‐PEP were smallest among all groups (Figure 4F–H), indicating that blocking ATAD3A phosphorylation with TAT‐PEP enhanced tumor sensitivity to doxorubicin. Consistently, SA‐β‐gal staining confirmed that doxorubicin‐induced tumor cell senescence was restrained by TAT‐PEP in vivo (Figure 4I; Figure S4F, Supporting Information). Tumor proliferation in both doxorubicin and doxorubicin combined with TAT treatments was decreased compared to the negative control (PBS) (Figure 4I; Figure S4F, Supporting Information), while doxorubicin in combination with TAT‐PEP further reduced the proliferation (Figure 4I; Figure S4F, Supporting Information). In contrast, TAT‐PEP enhanced doxorubicin‐induced tumor apoptosis, as indicated by the expression of cleaved caspase3 (Figure 4I; Figure S4F, Supporting Information). The inhibitory effect of TAT‐PEP on ATAD3A phosphorylation was confirmed by the IHC and WB results as shown in Figure S4G,H. Additionally, we detected higher level of mitophagy in the group treated with doxorubicin and TAT‐PEP than the groups of doxorubicin alone or combined with TAT (Figure 4J; Figure S4H, Supporting Information), suggesting that TAT‐PEP promoted mitophagy in vivo. Collectively, our findings reveal that targeting ATAD3A phosphorylation with TAT‐PEP in combination with chemotherapeutic agents may be a promising strategy to eliminate tumors.

### 2.5 TAT‐PEP Improves Physical Function and Ameliorates Aging‐Associated Signatures in Naturally Aged Mice

**Figure 5:**
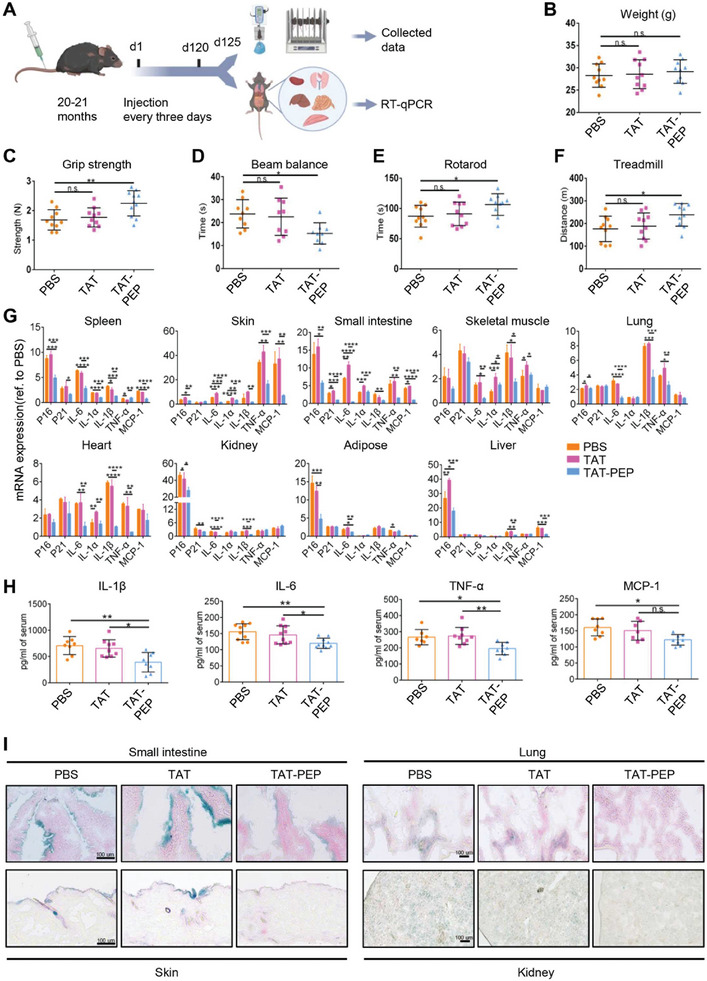
TAT‐PEP ameliorates aging‐associated signatures in naturally aged mice. A) Schematic diagram of the experimental design. Aged mice (20‐21 months) were divided into 3 groups treated with PBS, TAT, or TAT‐PEP. Tail vein injections of PBS (100 µL per mouse), TAT (1 mg mL^−1^, 100 µL per mouse), or TAT‐PEP (1 mg mL^−1^, 100 µL per mouse) were administered every three days for four months. Experiments were conducted 5 days after the last injection. B) Body weight of aged mice treated with PBS, TAT, or TAT‐PEP (*n =* 10 for each group). n.s., not significant (one‐way ANOVA with Tukey post‐hoc test). C) Grip strength of aged mice treated with PBS, TAT, or TAT‐PEP (*n =* 10 for each group). n.s., not significant, **p < 0.01 (one‐way ANOVA with Tukey post‐hoc test). D) Time to cross balance beam for old mice treated with PBS, TAT, and TAT‐PEP (*n =* 9 for each group, respectively). n.s., not significant, *p < 0.05 (one‐way ANOVA with Tukey post‐hoc test). E) Quantification of maximal time on the rotarod for aged mice treated with PBS, TAT, and TAT‐PEP (*n =* 10 for each group). n.s., not significant, *p < 0.05 (one‐way ANOVA with Tukey post‐hoc test). F) Exhaustion distance on a treadmill for aged mice treated with PBS, TAT, and TAT‐PEP (*n =* 10 for each group). n.s., not significant, *p < 0.05 (one‐way ANOVA with Tukey post‐hoc test). G) Expression levels of p16, p21, IL‐6, IL‐1α, IL‐1β, TNF‐α, and MCP‐1 analyzed by RT‐qPCR in various tissues from aged mice treated with PBS, TAT, TAT‐PEP (*n =* 3 for each group). *p < 0.05, **p < 0.01, ***p < 0.001, ****p < 0.0001 (one‐way ANOVA with Tukey post‐hoc test). H) Protein levels of IL‐1β (*n =* 7), IL‐6 (*n =* 10), TNF‐α (*n =* 7), and MCP‐1 (*n =* 8) in blood serum from aged mice treated with PBS, TAT, or TAT‐PEP, measured by ELISA. n.s., not significant, *p < 0.05, **p < 0.01 (one‐way ANOVA with Tukey post‐hoc test). I) SA‐β‐gal staining of small intestine, skin, lung, and kidney from aged mice treated with PBS, TAT, and TAT‐PEP (*n =* 3). Scale bars, 100 µm. All data are presented as the mean ± SD.

Cellular senescence is one of the prominent hallmarks of aging.^[^ ^45^ ^]^ To examine whether TAT‐PEP is beneficial for anti‐aging in vivo, we performed therapeutic experiments in naturally aged mice (20–21 months). These mice were treated with PBS, TAT, or TAT‐PEP through tail vein injections for four months (**Figure** **5**A). Despite that the body weights of mice in all three groups showed no significant differences (Figure 5B), TAT‐PEP treatment significantly improved grip strength, reduced beam balance crossing time, increased maximal rotarod time and treadmill endurance compared to PBS and TAT treatments (Figure 5C–F). These data suggest that blocking ATAD3A phosphorylation via TAT‐PEP improves the healthy condition of aged mice. We further assessed multiple senescence‐associated molecular markers including p16, p21, and SASP factors IL‐6/IL‐1α/IL‐1β/TNF‐α/MCP‐1. These markers were found to be upregulated in multiple tissues of aged mice (Figure S5A, Supporting Information). After TAT‐PEP treatment, we observed a general downregulation of these markers in the spleen, skin, small intestine, and skeletal muscle, and a decrease in 2–4 markers in the lung, heart, liver, adipose tissue, and kidney, compared to PBS and TAT treatments (Figure 5G). Moreover, serum analysis confirmed the systemic decrease of several SASP factors upon TAT‐PEP treatment (Figure 5H). Additionally, SA‐β‐gal staining revealed a reduction in SA‐β‐gal‐positive cells in the small intestine, skin, lung, and kidney tissues of aged mice treated with TAT‐PEP (Figure 5I), supporting the role of TAT‐PEP in suppressing senescence in naturally aged mice. These findings indicate that targeting ATAD3A phosphorylation by a synthetic peptide TAT‐PEP prevents cellular senescence and counteracts aging. Additionally, we conducted experiments to investigate the effects of TAT‐PEP in senescent cells. Our results showed that TAT‐PEP treatment in senescent WI‐38 cells (Passage 40) could not markedly decrease senescent marker expression (p21, p53, β‐gal) or increase proliferation (EDU staining) (Figure S5B,C, Supporting Information). Instead, continuous TAT‐PEP treatment during the senescence process from P25 to P40 slowed the progression of senescence and helped maintain cellular proliferation capacity (Figure S5B,C, Supporting Information). Our data suggest that TAT‐PEP delays senescence rather than reverses senescence, by inhibiting ATAD3A phosphorylation.

### 2.6 The Phosphorylation of ATAD3A Essentially Suppresses Pink1‐dependent Mitophagy via Enhancing Pink1 Mitochondrial Import

**Figure 6:**
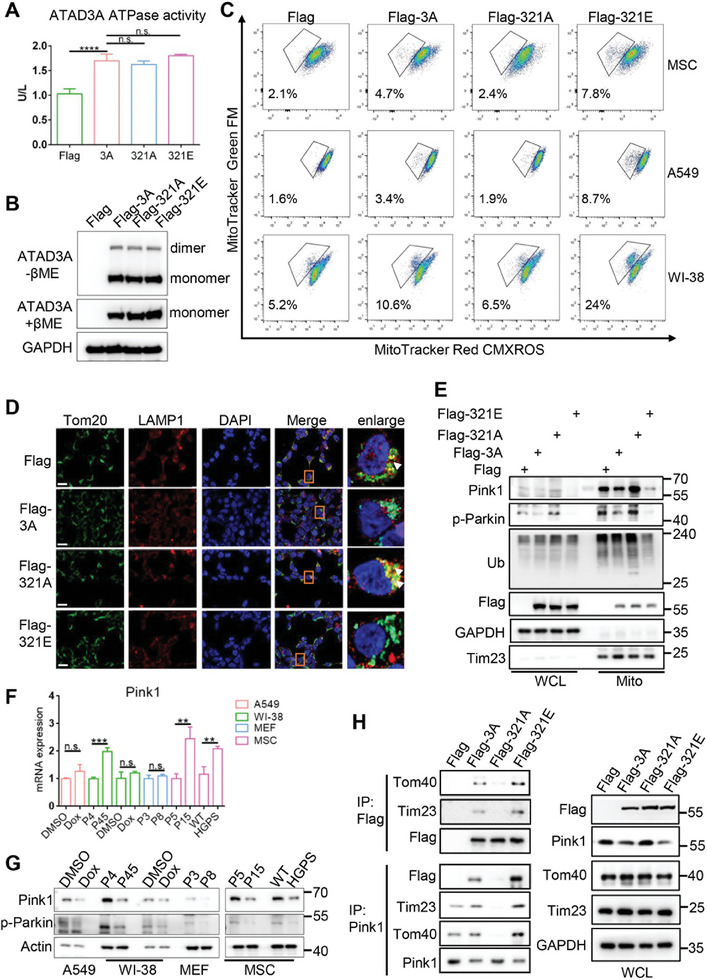
ATAD3A phosphorylation is required to suppress Pink1‐mediated mitophagy via enhancing Pink1 mitochondrial import. A) Detection of the ATPase activity of ATAD3A, ATAD3A S321A, and ATAD3A S321E in the presence of ATP (*n =* 3). ATAD3A and mutants were immunoprecipitated by using a Flag antibody. n.s., not significant, ****p < 0.0001 (one‐way ANOVA test with Tukey post‐hoc test). B) Immunoblot analysis of ATAD3A and mutant protein levels in A549 cells, in the presence or absence of β‐ME (*n =* 3). C) Flow cytometry was used to distinguish cell populations with high Mitotracker Green FM and low Mitotracker Red CMXROS (indicating damaged mitochondria) in MSC, A549, and WI‐38 cells transfected with Flag‐vector, Flag‐ATAD3A, Flag‐ATAD3A S321A or Flag‐ATAD3A S321E (*n =* 3). D) Confocal imaging of Tom20 (green) and LAMP1 (red) localization in 293T cells transfected with Flag‐vector, Flag‐ATAD3A, Flag‐ATAD3A S321A or Flag‐ATAD3A S321E (*n =* 3). Areas outlined by squares in the merged images are enlarged at right. Scale bars, 10 µm. E) Immunoblot analysis of Pink1, p‐Parkin, and Ubiquitin in the mitochondrial fraction of A549 cells overexpressing Flag‐vector, Flag‐ATAD3A, Flag‐ATAD3A S321A or Flag‐ATAD3A S321E (*n =* 3). F) The mRNA expression levels of Pink1 in non‐senescent and senescent A549, WI‐38, MEF, and MSC cells (*n =* 3). n.s., not significant, **p < 0.01, ***p< 0.001 (Unpaired two‐tailed T‐test). G) Immunoblot analysis of Pink1 in normal and senescent A549, WI‐38, MEF, and MSC cells (*n =* 3). H) Immunoblot analysis of the interaction of indicated proteins with ATAD3A or Pink1 in 293T cells transfected with Flag‐vector, Flag‐ATAD3A, or mutant ATAD3A (*n =* 3). All data are presented as the mean ± SD.

Given that phosphorylated ATAD3A is a druggable target for anti‐chemoresistance and anti‐aging treatments, we then investigated the downstream molecular mechanism of phosphorylated ATAD3A. The ATPase activity and oligomerization are important properties of ATAD3A and play crucial roles in mitochondrial homeostasis. We first tested whether the phosphorylation of ATAD3A influences its ATPase activity and oligomerization. As shown in **Figure** **6**A,B, neither ATAD3A S321A nor ATAD3A S321E changed the ATPase activity or oligomerization of ATAD3A compared to ATAD3A WT. The localization of ATAD3A in mitochondria also remained unchanged regardless of phosphorylation (Figure S6A, Supporting Information). On the other hand, we found that overexpression of ATAD3A caused a significant increase of damaged mitochondria mass compared to empty vector control and ATAD3A S321A, while overexpression of ATAD3A S321E showed more increase of damaged mitochondria mass compared to ATAD3A WT (Figure 6C; Figure S6B–D, Supporting Information). Consistently, the mitochondria membrane potential decreased in cells with ATAD3A WT overexpression and lower in cells with ATAD3A S321E overexpression (Figure S6E, Supporting Information).

Building on our previous study which showed ATAD3A acts as a suppressor in Pink1‐mediated mitophagy,^[^ ^24^ ^]^ and finding that blocking ATAD3A phosphorylation with TAT‐PEP significantly increased mitophagy (Figure 4J; Figure S4H, Supporting Information), we then investigated whether the phosphorylation of ATAD3A is essential for regulating mitophagy. Confocal microscopy revealed that mitochondria (Tom20) and lysosome (LAMP1) exhibited stronger co‐localization in cells transfected with ATAD3A S321A in contrast to the cells transfected with ATAD3A WT and ATAD3A S321E (Figure 6D), demonstrating the essentiality of ATAD3A phosphorylation in mitophagy inhibition. To further verify this notion, we examined the key regulators in Pink1‐mediated mitophagy pathway, including mitochondria localized Pink1, p‐Parkin, and ubiquitinated proteins. All of them were significantly increased in the mitochondrial fraction in cells transfected with ATAD3A S321A, while reduced in cells transfected with ATAD3A WT, and further decreased in cells transfected with ATAD3A S321E (Figure 6E; Figure S6F, Supporting Information). We also examined the Pink1 expression in cellular senescence. Although the mRNA levels of Pink1 were unchanged or upregulated in senescent cells compared to normal cells (Figure 6F), the protein levels of Pink1 and p‐Parkin in senescent cells were dramatically decreased (Figure 6G; Figure S6G, Supporting Information), negatively correlated with enhanced ATAD3A phosphorylation. Additionally, we observed that TAT‐PEP treatment effectively decreased the phosphorylation of ATAD3A and reduced the levels of Pink1, p‐Parkin, and ubiquitinated proteins in doxorubicin‐induced senescent cells (Figure S6H, Supporting Information), suggesting that TAT‐PEP's impact on mitophagy is closely related to the elevated ATAD3A phosphorylation during cellular senescence. These findings indicate that Pink1‐dependent mitophagy is suppressed by ATAD3A phosphorylation during the progression of senescence.

The transportation of Pink1 into mitochondria, followed by cleavage and degradation, is key for mitophagy inhibition. Pink1 mitochondrial import depends on both the mitochondrial outer‐membrane complex (TOM) and the mitochondrial inner‐membrane complex (TIM).^[^ ^46^ ^]^ Our previous study has demonstrated that ATAD3A serves as a bridging factor between TOM and TIM complex to mediate Pink1 mitochondrial import process.^[^ ^24^ ^]^ Co‐IP assays showed that phosphor‐dead mutant ATAD3A S321A barely interacted with Tom40 and Tim23, while phosphor‐mimicking mutant ATAD3A S321E increased interaction with Tim23 and Tom40 compared to ATAD3A WT (Figure 6H), suggesting ATAD3A phosphorylation is necessary for bridging TOM and TIM complex. Additionally, the interaction of ATAD3A, Tom40, and Tim23 with Pink1 was promoted by ATAD3A S321E mutation but abolished by ATAD3A S321A mutation (Figure 6H). Knocking down TBK1 to inhibit the phosphorylation of ATAD3A during cellular senescence also disturbed the interaction between ATAD3A and Tom40, as well as Tim23 (Figure S6I, Supporting Information). To test whether phosphorylation of ATAD3A specifically affects the mitochondrial import of Pink1, we constructed a GFP‐MTS fusion protein with a mitochondrial transport signal and found that its mitochondrial import was not affected by the overexpression of ATAD3A or its phosphorylation mutants (Figure S6J, Supporting Information). We further examined the transport of various mitochondrial import proteins (ANT1, PDH, and HSC70). Our result indicates that ATAD3A phosphorylation does not influence the mitochondrial import of ANT1, PDH, and HSC70 (Figure S6K, Supporting Information). Collectively, our data demonstrate that ATAD3A phosphorylation is required for facilitating Pink1 mitochondrial import to suppress mitophagy, leading to the accumulation of damaged mitochondria, which in turn, may result in cellular senescence.

### 2.7 The Switch from TBK1‐OPTN Axis to TBK1‐ATAD3A‐PINK1 Axis Promotes Cellular Senescence

**Figure 7:**
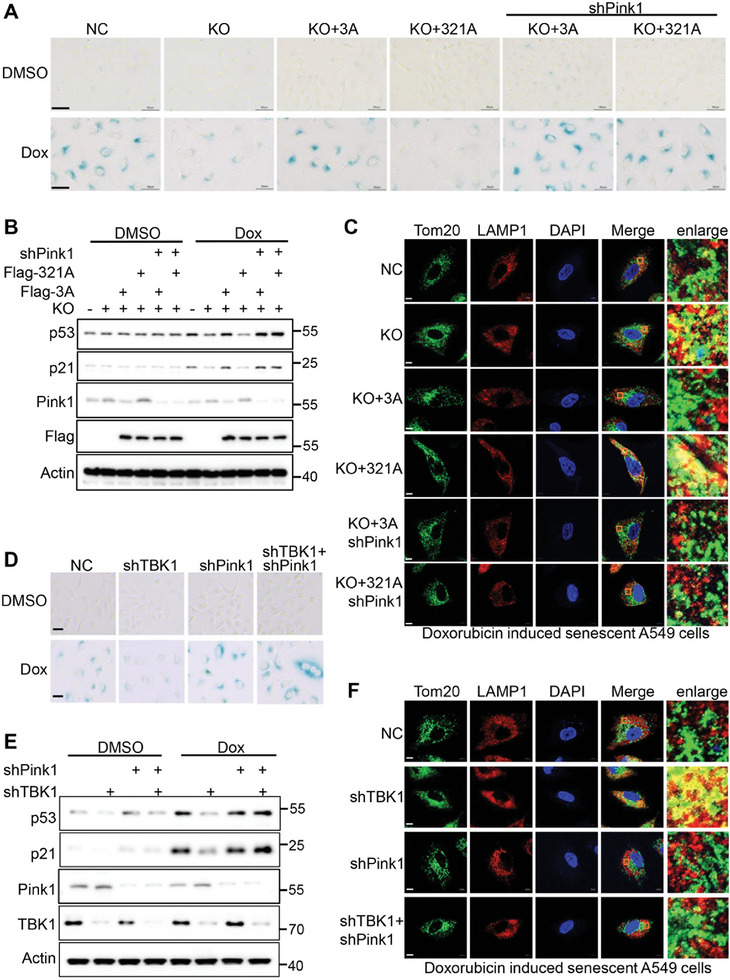
TBK1‐ATAD3A‐Pink1 axis inhibits mitophagy and promotes cellular senescence. A) SA‐β‐gal staining of indicated A549 cells (*n =* 3). Cells were initially induced with DMSO or doxorubicin (150 nm) for 48 h, followed by additional culturing for 48 h. B) Immunoblot analysis of p53, p21, and Pink1 in the cells described in A (*n =* 3). C) Confocal imaging of Tom20 (green) and LAMP1 (red) in indicated cells (*n =* 3). Cells were initially induced with doxorubicin (150 nm) for 48 h, followed by additional culturing for 48 h. Areas outlined by squares in the merged images are enlarged at right. Scale bars, 2 µm. D) SA‐β‐gal staining of NC, shTBK1, shPink1, and cells with combined shPink1 and shTBK1 (*n =* 3). The indicated A549 cells were initially induced with DMSO or doxorubicin (150 nm) for 48 h, followed by additional culturing for 48 h. E) Immunoblot analysis of p53, p21, and Pink1 in the cells described in D (*n =* 3). F) Confocal imaging of Tom20 (green) and LAMP1 (red) in NC, shTBK1, shPink1, and cells with combined shPink1 and shTBK1 (*n =* 3). Cells were initially induced with doxorubicin (150 nm) for 48 h, followed by additional culturing for 48 h. Areas outlined by squares in the merged images are enlarged at right. Scale bars, 2 µm.

Finally, we tested whether TBK1‐mediated ATAD3A phosphorylation promotes senescence via inhibiting Pink1‐dependent mitophagy. In line with the aforementioned results, ATAD3A deficiency repressed doxorubicin‐induced cellular senescence, which was rescued by restoring ATAD3A WT but not the restoration of phosphor‐dead mutant ATAD3A S321A accompanying Pink1 accumulation (**Figure** **7**A,B; Figure S7A,B, Supporting Information). Simultaneously knockdown of Pink1 recovered the senescence markers including SA‐β‐gal activity and p53, p21 expression (Figure 7A,B; Figure S7A,B, Supporting Information), indicating that Pink1 accumulation resulted from loss of ATAD3A phosphorylation inhibits cellular senescence. Consistently, Pink1 accumulation is responsible for elevated mitophagy in ATAD3A S321A restoration cells, as knocking down Pink1 reversed the increased mitophagy and reduced it to a similar level in ATAD3A S321A and ATAD3A cells (Figure 7C).

We further generated TBK1 knockdown A549 cell lines in combination with Pink1 knockdown. Consistent with the aforementioned results, TBK1 deletion reduced cellular senescence, as indicated by lower SA‐β‐gal activity and decreased expression of p53, and p21, compared to the negative control (NC). Simultaneously knockdown of Pink1 abolished the reduction of cellular senescence in TBK1 knockdown cells (Figure 7D,E; Figure S7C,D, Supporting Information). Consistently, TBK1 knockdown increased the activation of mitophagy upon doxorubicin senescence induction, but this effect was diminished when Pink1 was also knocked down (Figure 7F). These findings reveal that ATAD3A, its upstream kinase TBK1, and its downstream substrate Pink1 form a working axis to promote cellular senescence.

It's worth noting that previous reports have demonstrated that TBK1 facilitates mitophagy by phosphorylating autophagy adaptors such as OPTN,^[^ ^19^ ^]^ however, the relationship with senescence is unclear. Interestingly, the interaction between OPTN and TBK1 decreased in senescent cells (Figure S8A, Supporting Information). To further validate and define the specific interaction between ATAD3A and TBK1, we conducted co‐immunoprecipitation (co‐IP) assays using different fragments of ATAD3A and TBK1. Our results showed that the deletion of either the C‐terminal region (residues 677–729) of TBK1 or the transmembrane and mitochondrial signaling domain (residues 225–290) of ATAD3A abolished the formation of the ATAD3A/TBK1 complex (Figure S8B–D, Supporting Information). Notably, it has been reported that the C‐terminal region (residues 677–729) of TBK1 is necessary for interaction with OPTN.^[^ ^19^ ^]^ Our study revealed that the C‐terminal region (residues 677–729) of TBK1 is also critical for interaction with ATAD3A, supporting the notion that ATAD3A may competitively interact with TBK1, replacing OPTN, thereby leading to the inhibition of mitophagy and the acceleration of senescence. To further clarify whether the aberrant activation of TBK1 in senescent cells affects the shift from TBK1‐OPTN to TBK1‐ATAD3A, we constructed TBK1 phosphorylation mutants, including the S172A (phosphorylation‐dead) and S172E (phosphorylation‐mimicking) variants, and overexpressed them in A549 cells. We found that TBK1 phosphorylation is closely related to its mitochondrial localization, as the TBK1‐S172A level declined in isolated mitochondria (Figure S8E, Supporting Information). In senescent cells, TBK1‐S172A reduced the interaction between ATAD3A and TBK1 compared to TBK1‐WT, while TBK1‐S172E significantly increased this interaction (Figure S8F, Supporting Information). Conversely, the interaction between OPTN and TBK1 was found to be reduced with TBK1‐S172E and enhanced with TBK1‐S172A (Figure S8F, Supporting Information).

Overall, our study demonstrates that, during cellular senescence, TBK1 is abnormally activated and localizes to mitochondria to phosphorylate ATAD3A, thereby switching the TBK1‐OPTN mitophagy activation axis to TBK1‐ATAD3A‐Pink1 mitophagy inhibition axis to promote cellular senescence. Targeting ATAD3A phosphorylation by a synthetic peptide TAT‐PEP prevents cellular senescence, sensitizes tumors to chemotherapy, and counteracts aging (Figure S8G, Supporting Information).

## 3 Discussion

Eliminating or preventing senescent cells has emerged as a vital strategy for anti‐aging and anti‐tumor therapy. Therefore, thoroughly investigating the underlying mechanisms of cellular senescence and identifying modulators of senescence are important for the development of drugs targeting anti‐aging and age‐related diseases. In this study, we elucidate a switch of TBK1‐OPTN axis to TBK1‐ATAD3A‐Pink1 axis that promotes cellular senescence. We found that TBK1 localized to mitochondria and phosphorylated ATAD3A at Ser321 site, which enhanced Pink1 mitochondrial import to suppress mitophagy, resulting in damaged mitochondria accumulation, ultimately promoting cellular senescence. Notably, ATAD3A Ser321 phosphorylation, which markedly increases in senescent cells and various tissues of physiological and pathological aging mice, serves as a promising aging marker. Blocking Ser321 phosphorylation with a competitive peptide TAT‐PEP not only overcomes senescence‐associated chemoresistance of tumors but also ameliorates aging‐related signatures in naturally aged mice. Our findings give an extensive understanding of TBK1 and ATAD3A's function in chemoresistance and aging, providing potential strategies for anti‐chemoresistance anti‐aging therapy.

Although previous reports showed TBK1 negatively regulates senescence or aging‐related neurodegenerative diseases,^[^ ^14^, ^15^ ^]^ there is also evidence suggesting that TBK1 can promote cellular senescence^[^ ^47^ ^]^ and age‐related Alzheimer's disease (AD).^[^ ^16^ ^]^ These findings suggest that the regulation of TBK1 on senescence is multifaceted. In this study, we revealed a new mechanism of senescence that appears to be dependent on TBK1 activation rather than the expression level. Our results highlight that the abnormal activation and phosphorylation state of TBK1 play a key role in the process of senescence (Figure 1; Figure S8E, Supporting Information). Interestingly, we found that both OPTN and ATAD3A interacted with the C‐terminal region of TBK1 from previous^[^ ^48^ ^]^ and our study, suggesting OPTN and ATAD3A competitively interact with TBK1 to coordinately regulate mitophagy. Specifically, TBK1 phosphorylation in senescent cells promotes its interaction with ATAD3A rather than with OPTN (Figure 2B; Figure S8A, Supporting Information), thereby inhibiting mitophagy and accelerating cellular senescence. Collectively, these findings suggest a switch of TBK1‐OPTN‐mitophagy activation axis to TBK1‐ATAD3A‐mitophagy inhibition axis to drive senescence. Therefore, the findings of this study propose a new mechanism that explains TBK1's role in cellular senescence, which is not solely dependent on changes in its expression levels.

Mitophagy defects have been defined as one of the hallmarks of aging,^[^ ^45^ ^]^ but the underlying mechanism is largely unknown. The mitochondrial protein ATAD3A plays a vital role in maintaining mitochondrial homeostasis. In our previous study, we revealed that ATAD3A is a key suppressor of Pink1‐mediated mitophagy.^[^ ^24^ ^]^ Mutations or excess oligomerization of ATAD3A leads to mitochondrial dysfunction, which is closely associated with age‐related neurological symptoms, such as Huntington's disease and Alzheimer's disease.^[^ ^26^, ^27^, ^48^ ^]^ However, the role of ATAD3A in cellular senescence and aging remains unclear. Acetylation of ATAD3A has been identified to be associated with Huntington's disease,^[^ ^26^ ^]^ highlighting the significant role of post‐translational modifications in ATAD3A functions. In our study, we revealed that ATAD3A phosphorylation at the Ser321 sites facilitated its suppression of mitophagy, driving cellular senescence. These findings link mitophagy with cellular senescence and provide new insight into ATAD3A in cellular senescence. Multiple studies have revealed that ATAD3A acts as an anti‐apoptotic regulator and is involved in chemo‐resistance during tumor treatment, suggesting it is an attractive target in combination with chemotherapy.^[^ ^49^, ^50^, ^51^ ^]^ We show that a blocking peptide TAT‐PEP targeting ATAD3A phosphorylation prevented doxorubicin‐induced senescence and promoted tumor cell apoptosis, thereby sensitizing the tumor cells to chemotherapy. These data provide a high‐potential therapeutic strategy for cancer treatment and chemotherapy enhancement.

Several senolytic drugs such as dasatinib and quercetin, have shown effectiveness in the elimination of senescent cells and counteract senescence‐associated pathologies in various models of aging and age‐related diseases in humans.^[^ ^52^, ^53^ ^]^ Senomorphic agents like rapamycin and metformin are known to suppress SASP production and prevent cellular senescence, attenuating aging signatures.^[^ ^2^, ^11^ ^]^ Enhancing mitophagy by overexpressing mitophagy‐specific proteins or supplementing metabolites that specifically induce mitophagy has been shown to slow systematic aging and extend lifespan in flies and mice,^[^ ^54^, ^55^, ^56^, ^57^ ^]^ suggesting induction of mitophagy maybe an effective strategy to prevent cellular senescence and aging. Our study reveals Ser321 phosphorylation of ATAD3A, which acts as a suppressor of mitophagy, accelerates senescence, and serves as a druggable target. We define Ser321 phosphorylation blocking peptide TAT‐PEP to prevent cellular senescence, leading to improved physical function and attenuated age‐associated signatures in naturally aged mice. Thus, we develop a promising therapeutic strategy targeting senescent cells for anti‐aging therapy.

There are still some limitations to this study. For instance, the in vivo evaluation of TAT‐PEP in ameliorating senescence‐associated pathologies is performed in two mouse models of senescence‐related chemo‐resistance and physiological aging. The expending conclusion to other models requires further investigation in the future. Additionally, the stability and dosage of TAT‐PEP may be optimized to improve its efficiency. For in vivo cancer treatments, combining more chemotherapy drugs rather than doxorubicin with TAT‐PEP needs additional investigation.

Taken together, our study identifies a pro‐senescence axis, TBK1‐ATAD3A‐Pink1, and provides a potential clinical target for anti‐aging therapies and strategies to combat chemoresistance in tumor treatments.

## 4 Experimental Section

### Key Resources Table

**Table:** 

REAGENT or RESOURCE	SOURCE	IDENTIFIER
Antibodies		
ATAD3A	Abnova	Cat#H00055210; RRID:AB_10718149
p‐Ser/Thr	Abcam	Cat# ab17464; RRID:AB_443891
p21	Abcam	Cat# ab109199; RRID:AB_10861551
p16	Abcam	Cat# ab108349; RRID:AB_10858268
HA	Abcam	Cat# ab9110; RRID:AB_307019
LAMP1	Abcam	Cat# ab25630; RRID:AB_470708
Ki‐67	Abcam	Cat# ab15580; RRID:AB_443209
VeriBlot for IP Detection Reagent (HRP)	Abcam	Cat# ab131366; RRID:AB_2892718
p‐Parkin	Abcam	Cat# ab ab315376
HSC‐70	Abcam	Cat# ab19136 , RRID:AB_444764
TBK1	CST	Cat# 38 066; RRID:AB_2827657
PLK1	CST	Cat# 4513; RRID:AB_2167409
PKCδ	CST	Cat# 9616; RRID:AB_10949973
Cleaved caspase3	CST	Cat# 9664; RRID:AB_2070042
PKM2	CST	Cat# 4053; RRID:AB_1904096
PDH	CST	Cat# 3205; RRID:AB_2162926
GAPDH	CST	Cat# 2118; RRID:AB_561053
Tom20	Santa Cruz	Cat# sc‐11415; RRID:AB_2207533
Tim23	Santa Cruz	Cat# sc‐514463; RRID:AB_2923126
Tom40	Santa Cruz	Cat# sc‐365467; RRID:AB_10847086
GFP	Santa Cruz	Cat# sc‐9996; RRID:AB_627695
TBK1	Santa Cruz	Cat# sc‐398366
β‐Actin	Santa Cruz	Cat# sc‐8432; RRID:AB_626630
Ubiquitin	Santa Cruz	Cat# sc‐8017; RRID:AB_628423
OPTN	Santa Cruz	Cat# sc‐166576; RRID:AB_2156554
p53	Santa Cruz	Cat# sc‐126; RRID:AB_628082
Flag	Sigma	Cat# F3165; RRID:AB_259529
Mitofilin	Novus	Cat#NB100‐74461
ANT1	Proteintech	Cat# 30631‐1‐AP
Goat anti‐Rabbit IgG (H+L) Secondary Antibody, HRP	Thermo Fisher	Cat# 31460
Goat anti‐Mouse IgG (H+L) Secondary Antibody, HRP	Thermo Fisher	Cat# 31430
Goat anti‐Rabbit IgG (H+L) Secondary Antibody, Alexa Fluor 488	Thermo Fisher	Cat# A‐11008; RRID:AB_143165
Goat anti‐Mouse IgG (H+L) Secondary Antibody, Alexa Fluor 488	Thermo Fisher	Cat# A‐11001; RRID:AB_2534069
Donkey anti‐Rabbit IgG (H+L) Secondary Antibody, Alexa Fluor 594	Thermo Fisher	Cat# A‐21207; RRID:AB_141637
Goat anti‐Rabbit IgG (H+L) Secondary Antibody, Alexa Fluor 594	Thermo Fisher	Cat# A‐11037; RRID:AB_2534095
Chemicals		
Doxorubicin	Selleck	Cat# E2516
Recombinant TBK1 Protein	Abcam	Cat# ab85276
Protease Inhibitor Cocktail	Roche	Cat# P8340
Phosphatase inhibitor cocktail	Roche	Cat# PHOSS‐RO
N‐ethylmaleimide	MCE	Cat# HY‐D0843
Protein A/G agarose	Santa Cruz	Cat# sc‐2003
MitoTracker Green FM	Thermo Fisher	Cat# M7514
MitoTracker CMXROS	Thermo Fisher	Cat# M46752
MitoTracker Deep Red FM	Thermo Fisher	Cat# M22426
REAGENT or RESOURCE	SOURCE	IDENTIFIER
Plasmids		
PCNDA3.1‐HA	Addgene	Cat# 128 034; RRID:Addgene_128034
PCNDA3.1‐eGFP	Addgene	Cat# 129 020;
PCNDA3.1‐Flag	Addgene	Cat# 208 051; RRID:Addgene_208051
PCDH‐pCDH‐CMV‐MCS‐EF1‐Puro	Novopro	Cat# V006738
LentiCRISPRv2	Addgene	Cat# 98 290; RRID:Addgene_98290
PLKO.1‐blast	Addgene	Cat# 26 655; RRID:Addgene_26655
PLKO.1‐neo	Addgene	Cat# 13 425; RRID:Addgene_13425
Critical commercial assays		
QuikChange Site‐Directed Mutagenesis Kit	Stratagene	Cat# 200518
Live & Dead Viability/Cytotoxicity Assay Kit	Keygen Biotech	Cat# KGAF001
kFlour488‐EdU Cell Proliferation Detection Kit	Keygen Biotech	Cat# KGA9602
Mitochondria Isolation Kit	Keygen Biotech	Cat# KGA3106
Senescent Cells and Tissues Staining Kit	Beyotime Biotechnology	Cat# RG0039
Mitochondrial Membrane Potential Detection Kit	Beyotime Biotechnology	Cat# C2006
HiPure Universal RNA Mini Kit	Magen	Cat# R4130
PrimeScript One Step RT‐PCR Kit	Takara	Cat# RR037A
ChamQ Universal SYBR qPCR Master Mix	Vazyme	Cat# Q711‐02
Mouse Interleukin 1β (IL‐1β) ELISA Kit	Elabscience	Cat# E‐MSEL‐M0003
Mouse Interleukin 6 (IL‐6) ELISA Kit	Elabscience	Cat# E‐MSEL‐M0001
Mouse Tumor Necrosis Factor α (TNFα) ELISA Kit	Elabscience	Cat# E‐EL‐M3063
Mouse Monocyte Chemotactic Protein 1 (MCP‐1) ELISA kit	Elabscience	Cat# E‐MSEL‐M0012
Biological samples		
C57BL/6 mouse tissue sections	In house procedure	For information contact Guoxian Jin
HGPS(*Lmna^609^*) mouse tissue sections	In house procedure	For information contact Guoxian Jin
Experimental models: Mouse Strain		
BALB/c Nude female mice	Charles River	Cat# 401
C57BL/6J mice (20‐21 months)	Jackson Laboratory	Strain# 000664 RRID:IMSR_JAX:000664
Software and algorithms		
GraphPad Prism 6.0	GraphPad Software	https://www.graphpad.com/
FlowJo v7	FlowJo, LLC	https://www.flowjo.com
ImageJ	ImageJ	https://imagej.net/ij/
Other		
LSM900 laser‐scanning confocal microscope	ZESS	N/A
Transmission electron microscopy	FEI Company	N/A
CytoFLEX LX	Beckman	N/A
qTOWER^3^ for qPCR	Analytik Jena	N/A

### Animals

The young (3 months), and old (20‐21 months) male C57BL/6J mice were obtained from the Jackson Laboratory. BALB/c nude mice used for tumor xenografts were purchased from Charles River. All mouse experiments were performed under protocols approved by the Ethics Committee of Guangdong Provincial People's Hospital (KY2023‐399‐01).

### Cell Culture and Senescence Induction

HEK293T was cultured in DMEM (Hyclone, Logan, UT) supplemented with 10% FBS and 1% antibiotics (100 unit mL^−1^ penicillin, 100 µg mL^−1^ streptomycin). Human embryonic fibroblasts WI‐38 were cultured in EBSS/MEM (Hyclone, Logan, UT) supplemented with 10% FBS, and 1% antibiotics (100 unit mL^−1^ penicillin, 100 ug mL^−1^ streptomycin). For generating a replicative senescence model, WI‐38 cells were split 1:4 by seeding 1 × 10^6^ cells/100 cm^2^ culture dish until they entered senescence after ≈45 cumulative populations. For doxorubicin‐induced senescence, cells were treated with doxorubicin (150 nm mL^−1^) for 3 days, followed by 3 days of further culture in a drug‐free medium. Primary mouse embryonic fibroblasts (MEFs, second passage) were cultured in DMEM supplemented with 10% FBS and 1% antibiotics (100 unit mL^−1^ penicillin, 100 ug mL^−1^ streptomycin). Cells were split 1:3 until entering senescence after ≈7–8 cumulative populations. H1‐MSCs used for generating replicative senescence model, WT‐hMSCs and HGPS‐hMSCs (HGPS), which has been described previously,^[^ ^42^ ^]^ were cultured in DMEM (Hyclone, Logan, UT) supplemented with 10% FBS, 2 mm glutamine, 1% antibiotics (100 unit mL^−1^ penicillin, 100 ug mL^−1^ streptomycin). H1‐MSCs were split 1:4 until entering senescence after ≈12–15 cumulative populations. The human lung adenocarcinoma cells A549 were cultured in McCoy's 5 A medium supplemented with 10% FBS and 1% antibiotics (100 unit mL^−1^ penicillin, 100 ug mL^−1^ streptomycin). For doxorubicin‐induced senescence, cells were treated with doxorubicin (150 nm mL^−1^) for 48 h, followed by 48 h further culture in a drug‐free medium. All of the above cells were maintained at 37 °C in an incubator with 5% CO_2_‐95% air condition.

### Antibodies and Reagents

All antibodies, reagents, kits, and other resources used in this study were listed in the key resource table.

### Plasmids

The cDNA encoding ATAD3A was amplified by PCR from 293T cDNA library and was cloned into the vectors pcDNA3.1‐FLAG, PCDH‐pCDH‐CMV‐MCS‐EF1‐Puro. The cDNA encoding TBK1 was amplified by PCR from 293T cDNA library and was cloned into the vector pcDNA3.1‐HA. The truncated segments of ATAD3A and TBK1 were separately cloned into pcDNA3.1‐eGFP or pcDNA3.1‐HA. Specific point mutations of ATAD3A were introduced by site‐directed mutagenesis with a QuikChange Site‐Directed Mutagenesis Kit (Stratagene). The primers of ATAD3A mutations were listed in Table S1, Supporting Information.

### Anti‐Ser321 Phosphorylated ATAD3A Antibody Generation

The peptide (LS(p)PSLEARVRDIA‐C) conjugated with KLH was used to immunize New Zealand White rabbits. After five immunizations, the generated antibody was purified through affinity purification.

### Immunoblot, Immunoprecipitation, and Immunofluorescence Analysis

Immunoblot, immunoprecipitation, and immunofluorescence were conducted as previously described with minor modifications.^[^ ^24^ ^]^ Briefly, the cells or mitochondria isolated by using a Mitochondria Isolation Kit (Keygen Biotech) were lysed in RIPA buffer containing protease inhibitor cocktail (Roche), phosphatase inhibitor cocktail (Roche), and N‐ethylmaleimide (NEM). Tissues collected from mice were homogenized in RIPA buffer containing protease and phosphatase inhibitors. Then the lysates were subjected to immunoblot analysis with primary antibodies. For immunoprecipitation, whole‐cell lysates were incubated overnight at 4 °C with primary antibodies, and then incubated with protein A/G agarose beads for an additional 4 h at 4 °C. The beads were then washed four times with lysis buffer and subjected to immunoblot analysis. For immunofluorescence staining, cells were seeded in the confocal chamber, then fixed with 4% PFA, permeabilized with 0.2% TritonX‐100, and stained with the indicated antibodies. Frozen‐cut tissue slices were washed with PBS, and fixed with 4% PFA, permeabilized with 0.5% TritonX‐100 and stained with the indicated antibodies. Images were obtained with a ZEISS LSM900 laser‐scanning confocal microscope equipped with a 100× objective lens with laser excitation at 405, 488, and 568 nm.

### SA‐β‐gal Staining

SA‐β‐gal staining was conducted using the Senescent Cells and Tissues Staining Kit (Beyotime Biotechnology), according to the manufacturer's protocols. Briefly, cells seeded in six‐well plates or frozen tissue slices were washed with PBS and then fixed with the fixative solution for 10–15 minutes at room temperature. After fixation, the samples were washed 2–3 times with PBS. The staining solution was prepared freshly before use (the concentrations and components referred to the manufacturer's protocols). The samples were incubated with the staining solution at 37 °C (no CO_2_) overnight. The incubation time may vary depending on the sample and should be optimized. After incubation, the cells or tissue sections were washed with PBS and the images were taken with a light microscope. Senescent cells typically exhibited blue staining in the cytoplasm.

### CRISPR/Cas9 Gene Editing

LentiCRISPRv2 which contains Cas9 was used to delete an ATAD3A genomic fragment in the A549 cell line. The gRNA targeting ATAD3A sequence: F‐CACCGCGGCGGTGCGAGCATGTCG, R‐AAACCGACATGCTCGCACCGCCGC, were inserted into the LentiCRISPRv2. Cells in a logarithmic growth phase were transfected with the plasmid encoding gRNA by nucleofection (Lonza 4D Nucleofector system). 48 h after transfection, 1 µg mL^−1^ puromycin was used to select the transfected cells. After the selection, cells were sorted on a flow cytometer (BD FACS Aria II), and a single cell was seeded into each well of the 96‐well plates. The ATAD3A genome‐edited cell line was identified by genomic DNA sequencing.

### Construction of Stable Knockdown Cell Lines

pLKO.1‐shTBK1‐neo or pLKO.1‐shPink1‐bla, PLP1, PLP2, VSVG 3‐pack system were used for packaging the expression‐silencing viruses. The viruses were purified using Lentivirus Precipitation Solution (TransGen Biotech) and then used to infect A549 cells. After 12 h, the medium was replaced with a fresh complete medium. After 48 h, 10 µg mL^−1^ G418 or 8 µg mL^−1^ Blasticidin were used to select the infected cells. The sequences of the shRNAs against TBK1 and Pink1 were listed in Table S2, Supporting Information.

### Mass Spectrometric Analysis

293T cells transfected with HA‐TBK1 or Flag‐ATAD3A were lysed, followed by IP with anti‐HA or anti‐Flag antibody. The purified total IP HA product or Flag‐ATAD3A protein was resuspended in Laemmli loading buffer, boiled, and subsequently separated by SDS–PAGE. The gels were excised and subjected to tryptic digestion. Following desalting, the resultant peptides were analyzed through tandem mass spectrometry.

### Measurement of Mitochondrial Mass, Membrane Potential

For measurement of mitochondrial mass, cells were incubated with 50 nm MitoTracker Green FM and 100 nm MitoTracker CMXROS at 37 °C for 30 min, and analyzed by flow cytometry. For the measurement of membrane potential, the Mitochondrial Membrane Potential Detection Kit (Beyotime Biotechnology) was used according to the manufacturer's protocols.

### Mitochondrial Isolation and Mitochondrial Protease Protection Assay

Mitochondrial isolation was conducted using a Mitochondria Isolation Kit (Keygen Biotech) according to the manufacturer's protocols. The mitochondrial protease protection assay was performed based on a previous report^[^ ^58^ ^]^ with minor modifications. Briefly, isolated mitochondria were divided into four aliquots and subjected to the following treatments: 1) one aliquot was re‐suspended in Solution A (250 mm sucrose, 5 mm NaN_3_, 2 mm EGTA, 20 mm HEPES‐Na, pH 7.4) and incubated on ice for 8 min (untreated mitochondria); 2) one aliquot was re‐suspended in Solution A containing 20 µg mL^−1^ of PK and incubated on ice for 8 min (for digestion of surface‐exposed OMM proteins); 3) one aliquot was subjected to osmotic shock (OS) by re‐suspension in a hypotonic solution (5 mm HEPES, 5 mm sucrose, 1 mm EGTA, pH 7.4) on ice for 10 min (to disrupt the OMM), followed by the addition of an equal volume of hypertonic solution (750 mm KCl, 80 mm HEPES, 1 mm EGTA, pH7.4) containing 20 µg mL^−1^ PK for 10 min (for digestion of surface‐exposed OMM/IMM proteins); 4)one aliquot was re‐suspended in Solution A with 1% (v/v) Triton X‐100 (Tx‐100) and 20 µg mL^−1^ of PK (for digestion of all mitochondrial proteins). Finally, the mitochondria were pelleted by centrifugation at 10 000 g for 10 min at 4 °C.

### Immunohistochemistry

Paraffin‐embedded tissue sections were deparaffinized in xylene and rehydrated through graded alcohols to water. After antigen retrieval, endogenous peroxidase blocking, and non‐specific binding blocking, the sections were stained with the indicated primary antibodies, followed by incubation with secondary antibodies. The sections were then counterstained with hematoxylin, followed by dehydration and mounting. Images were then captured using a light microscope.

### Immunoelectron Microscopy

Samples for immunoelectron microscopy were prepared as previously described with minor modifications.^[^ ^24^ ^]^ In brief, cells were fixed and subsequently frozen for sectioning. After blocking, they were incubated with a primary antibody (TBK1 antibody) followed by incubation with a gold‐labeled secondary antibody. Sections were then stained with 2% uranyl acetate and lead citrate to enhance contrast under the electron microscope. Images were captured using an electron microscope (JEM1400, JEOL).

### RT‐qPCR

Total RNA was isolated using the HiPure Universal RNA Mini Kit (Magen). The mRNA was treated with DNase and then converted into cDNA using the PrimeScript One Step RT‐PCR Kit (Takara). RT‐qPCR was performed using ChamQ Universal SYBR qPCR Master Mix (Vazyme) according to the manufacturer's protocols. Actin was used as a control to normalize the expression of target genes. Primers for specific genes of mouse and humans were listed in Table S3, Supporting Information.

### Enzyme‐linked Immunosorbent Assay

Mouse blood samples were clotted overnight at 4 °C, and then centrifuged (3000 rpm for 10 min) to collect serum. Mouse IL‐1β, IL‐6, TNFα, and MCP‐1 were measured using Mouse Interleukin 1β (IL‐1β) ELISA Kit (Elabscience), Mouse Interleukin 6 (IL‐6) ELISA Kit (Elabscience), Mouse Tumor Necrosis Factor α (TNFα) ELISA Kit (Elabscience), Mouse Monocyte Chemotactic Protein 1 (MCP‐1) ELISA kit (Elabscience) according to the manufacturer's protocols.

### Animal Experiments

A549 cells were collected and resuspended in PBS. The tumor xenografts were established by injecting 2 × 10^6^ cells into the subcutaneous per mouse. When the tumor size reached nearly 80–100 mm^3^, the doxorubicin treatment was started. Doxorubicin (5 mg kg^−1^) alone or doxorubicin combined with TAT and TAT‐PEP (100 µL, 1 mg mL^−1^) were injected into the tail vein according to the indicated time intervals. After 1.5 months, the mice were euthanized and tumors were isolated for imaging and measurement.

For the treatment of aged mice, the mice (20–21 months) were divided into three groups: PBS, TAT, and TAT‐PEP treated groups. PBS (100 µL), TAT (100 µL, 1 mg mL^−1^), or TAT‐PEP (100 µL, 1 mg mL^−1^) were injected into the tail vein per mouse every three days. After four months, the physical function and RT‐qPCR measurements were performed.

### Physical Function Measurements of Mice

All measurements were prepared and conducted according to a previously published report with minor modifications.^[^ ^59^ ^]^ Measurements were conducted at 5 days following the final dose of peptide injection. The mice were pre‐trained before the actual test. For the grid strength test, mice were positioned atop a grid strength meter. The grip strength was measured across three trials. Results were averaged from three trials.

For the beam balance test, the mice were trained to traverse the beam from one end to the other of the beam. On the test day, the mice were positioned on the start end and allowed to traverse the beam toward the opposite end. The time was recorded and the results were averaged from three trials.

For the rotarod test, each mouse was initially positioned on a rod rotating at 4 rpm, which then steadily accelerated to a top speed of 40 rpm over 5 min until the mouse fell off or gripped and rotated for two consecutive revolutions without walking. The time was recorded and the results were averaged from three trials.

For the treadmill exhaustion test, the mice underwent 3 days of training on a treadmill set at a 5° incline with 0.5 mA electrical stimulation, to acclimate them to the equipment. The training regimen began at a speed of 5 m min^−1^ for 2 min, then increased to 7 m min^−1^ for another 2 min, followed by 9 m min^−1^ for 1 min. On the test day, the mice started at 5 m min^−1^ for 2 min, with the speed increasing by 2 m min^−1^ every 2 min until the mice could no longer return to the treadmill. The distance was recorded and the results were averaged from 3 trials.

### Cell Viability, Death, and Proliferation Analysis

Cell viability and death were conducted using a Live & Dead Viability/Cytotoxicity Assay Kit (Keygen Biotech) according to the manufacturer's protocols. Cell proliferation was detected using a kFlour488‐EdU Cell Proliferation Detection Kit (Keygen Biotech) according to the manufacturer's protocols.

### Statistical Analysis

Statistical analyses and graphs were performed by using Prism (GraphPad 6.0 Software). Statistical significance was determined by a two‐tailed, unpaired Student's t‐test (for two groups comparing) or one‐way ANOVA with Dunnet or Tukey post‐hoc test (for more than two groups comparing). Statistical data were presented as mean ± standard error of the mean ± s.d. (SD). *p <*0.05 was considered statistically significant. * Indicates *p <*0.05; ** indicates *p <*0.01; *** indicates *p <*0.001; **** indicates *p* < 0.0001. The sample sizes (*n*) were provided in the figures and figure legends. Data were independent biological replicates. All quantifications and statistics have already been documented in the figure legend.

## Conflict of Interest

The authors declare no conflict of interest.

## Author Contributions

Y.H., Z.Z., and G.J. designed the experiments and analyzed the data; Y.H., Y.L., M.Z., Y.Z., and M.H. performed the experiments; L.W., G.G., and Z.Z. provided technical support, critical comments, and suggestions; Y.H. wrote the manuscript; G.J. reviewed, edited, and supervised the project.

## Supporting information



Supporting Information

Supporting Table 1

## Data Availability

The data that support the findings of this study are available from the corresponding author upon reasonable request.
