# Supplementary material for: Targeting ATAD3A Phosphorylation Mediated by TBK1 Ameliorates Senescence‐Associated Pathologies
Source: Adv Sci (Weinh). 2024 Nov 8;12(1):2404109. doi: 10.1002/advs.202404109 (PMC11714148; doi:10.1002/advs.202404109)
Supplement: Supplementary file 1 — Supporting Information [file ADVS-12-2404109-s002.pdf]

ADVANCED SCIENCE
Open Access

Supporting Information

for *Adv. Sci.*, DOI 10.1002/advs.202404109

Targeting ATAD3A Phosphorylation Mediated by TBK1 Ameliorates Senescence-Associated Pathologies

*Yujiao He, Yanchen Liu, Mingyue Zheng, Yuxiu Zou, Mujie Huang, Linsheng Wang, Ge Gao, Zhongjun Zhou and Guoxiang Jin**

Supporting Information

Targeting ATAD3A Phosphorylation Mediated by TBK1 Ameliorates Senescence-Associated Pathologies

Yujiao He, Yanchen Liu, Mingyue Zheng, Yuxiu Zou, Mujie Huang, Linsheng Wang, Ge Gao, Zhongjun Zhou, and Guoxiang Jin*

This file includes:
Figure S1 to S8
Table S1 to S3

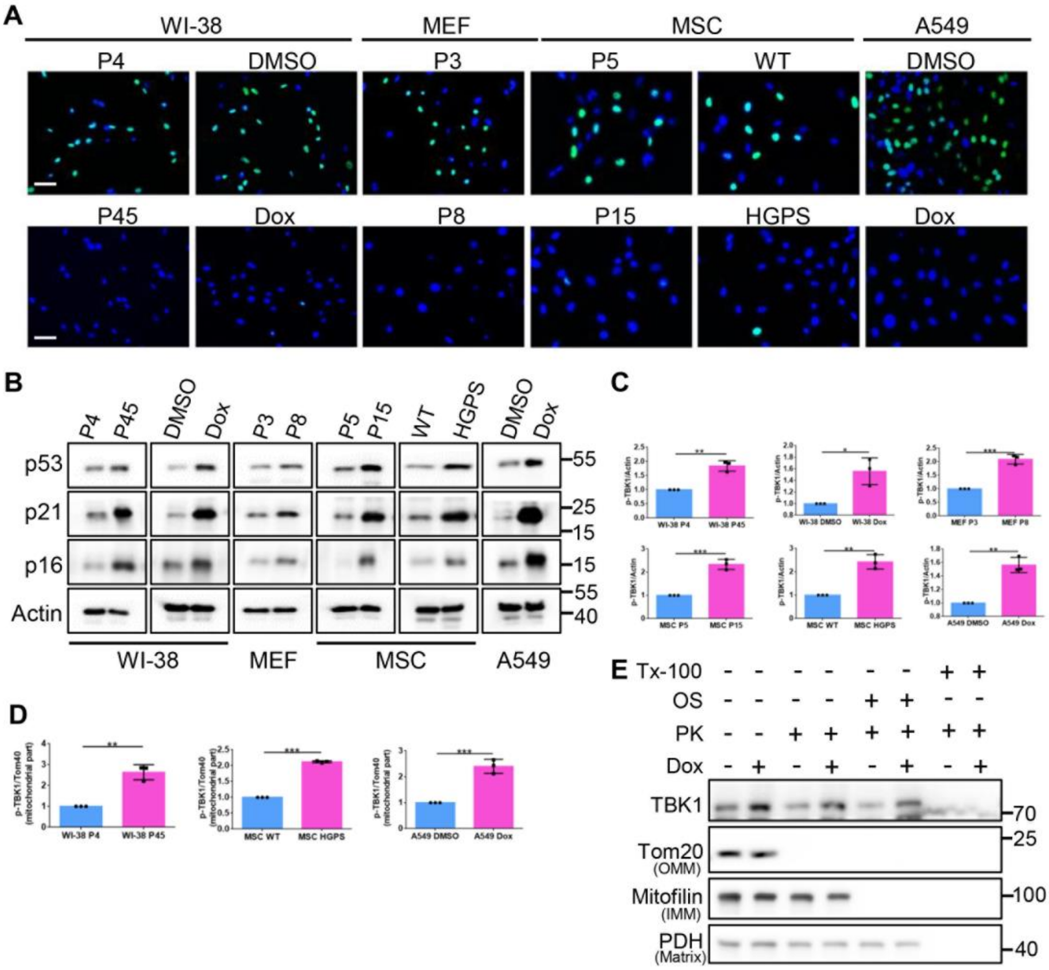

Figure S1. Aberrant TBK1 phosphorylation and localization in senescent cells. (A) Detection of cell proliferation of normal and senescent cells using EDU detection kit (n=3). Scale bars, 10 μm. (B) Immunoblot analysis of cellular senescent markers p53, p21, and p16 in normal and senescent cells (n=3). (C) Quantification of the immunoblot analysis as presented in Figure 1B (n=3). *P < 0.05, **P < 0.01, ***P < 0.001 (Unpaired two-tailed T-test). (D) Quantification of the immunoblot analysis as presented in Figure 1E (n=3). **P < 0.01, ***P < 0.001 (Unpaired two-tailed T-test). (E) Immunoblot analysis of TBK1 mitochondrial distribution in doxorubicin-induced senescent A549 cells using a mitochondrial protease protection assay (n=3). All data are presented as the mean ± SD.

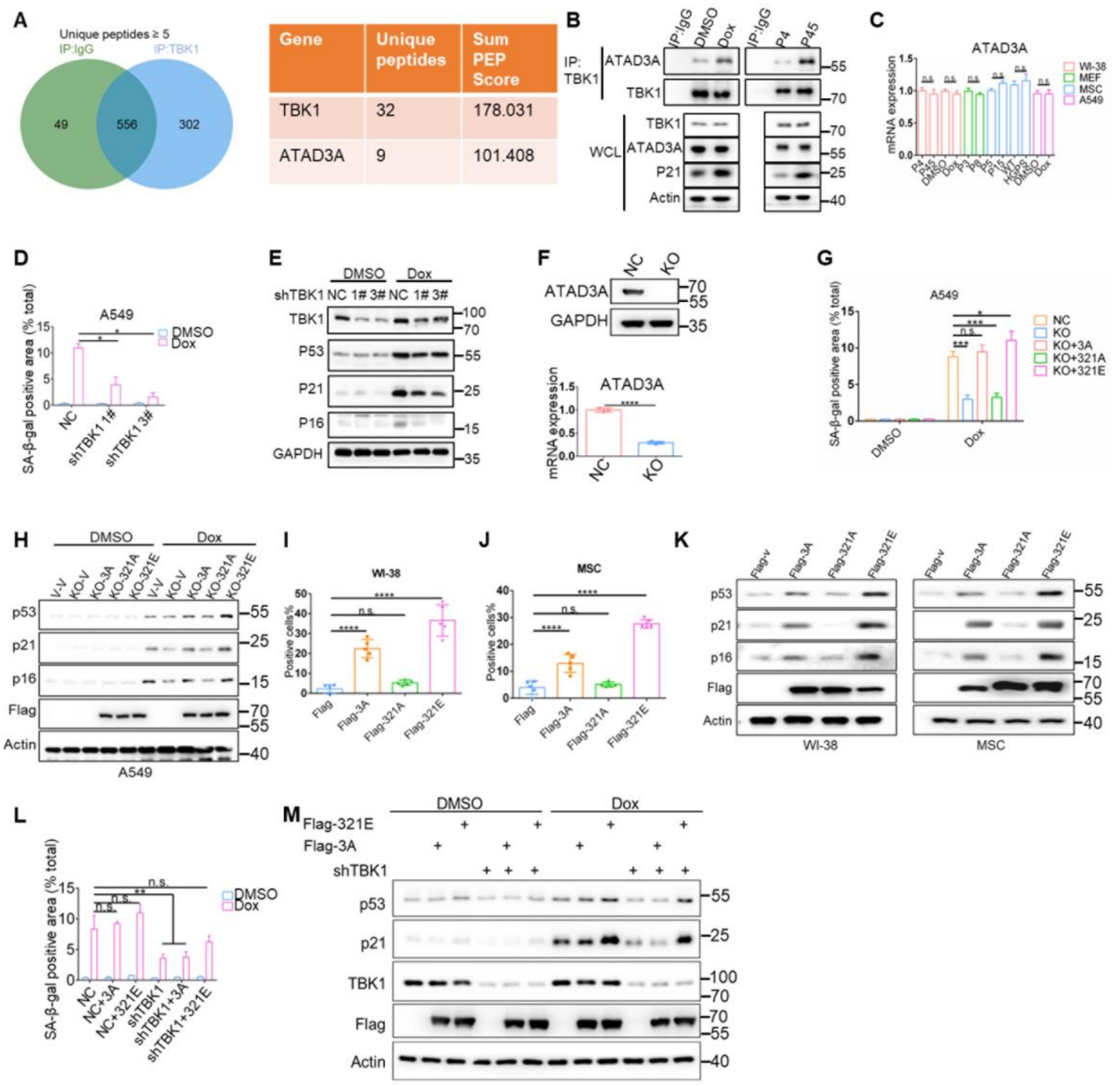

Figure S2 TBK1 phosphorylates ATAD3A to promote cellular senescence. (A) TBK1's interaction with ATAD3A was identified through MS analysis (n=1). (B) Immunoblot analysis of the interaction between endogenous ATAD3A and TBK1 by immunoprecipitating TBK1 from whole cell lysates in normal or senescent A549 and WI-38 cells (n=3). (C) The mRNA expression levels of ATAD3A in normal and senescent cells. n.s., not significant (Unpaired two-tailed T-test) (n=3). (D) Quantification of SA-β-gal staining as presented in Figure 2I (n=3). Analysis was based on at least three different images covering dozens of cells using the ImageJ software. *P < 0.05 (one-way ANOVA with Dunnet post-hoc test). (E) Immunoblot analysis of p53, p21, p16 in the cells described in Figure 2I (n=3). (F) Generating ATAD3A knockout (KO) A549 cell lines using CRISPR editing. Immunoblot analysis of ATAD3A expression in NC (negative control) and ATAD3A (KO) cells (upper) (n=3). The mRNA expression levels of ATAD3A in NC and ATAD3A (KO) A549 cells were shown below. ****P<0.0001 (Unpaired two-tailed T-test). (G) Quantification of SA-β-gal staining as presented in Figure 2J (n=3). Analysis was based on five different images covering dozens of cells using the ImageJ software. n.s., not significant, *P < 0.05, ***P < 0.001 (one-way ANOVA with Dunnet post-hoc test). (H) Immunoblot analysis of cellular senescent markers p53, p21, p16 in cells described in Figure 2J (n=3). (I, J) Quantification of SA-β-gal staining as presented in Figure 2K (n=3). Analysis was based on five different images covering dozens of cells using the ImageJ software. n.s., not significant, ****P < 0.0001 (one-way ANOVA with Dunnet post-hoc test). (K) Immunoblot analysis of cellular senescent markers p53, p21, p16 in cells described in Figure 2K (n=3). (L)

Quantification of SA-β-gal staining as presented in Figure 2L (n=3). Analysis was based on at least three different images covering dozens of cells using the ImageJ software. n.s., not significant, $**P < 0.01$ (one-way ANOVA with Dunnet post-hoc test). (M) Immunoblot analysis of cellular senescent markers p53, p21, p16 in cells described in Figure 2L (n=3). All data are presented as the mean ± SD.

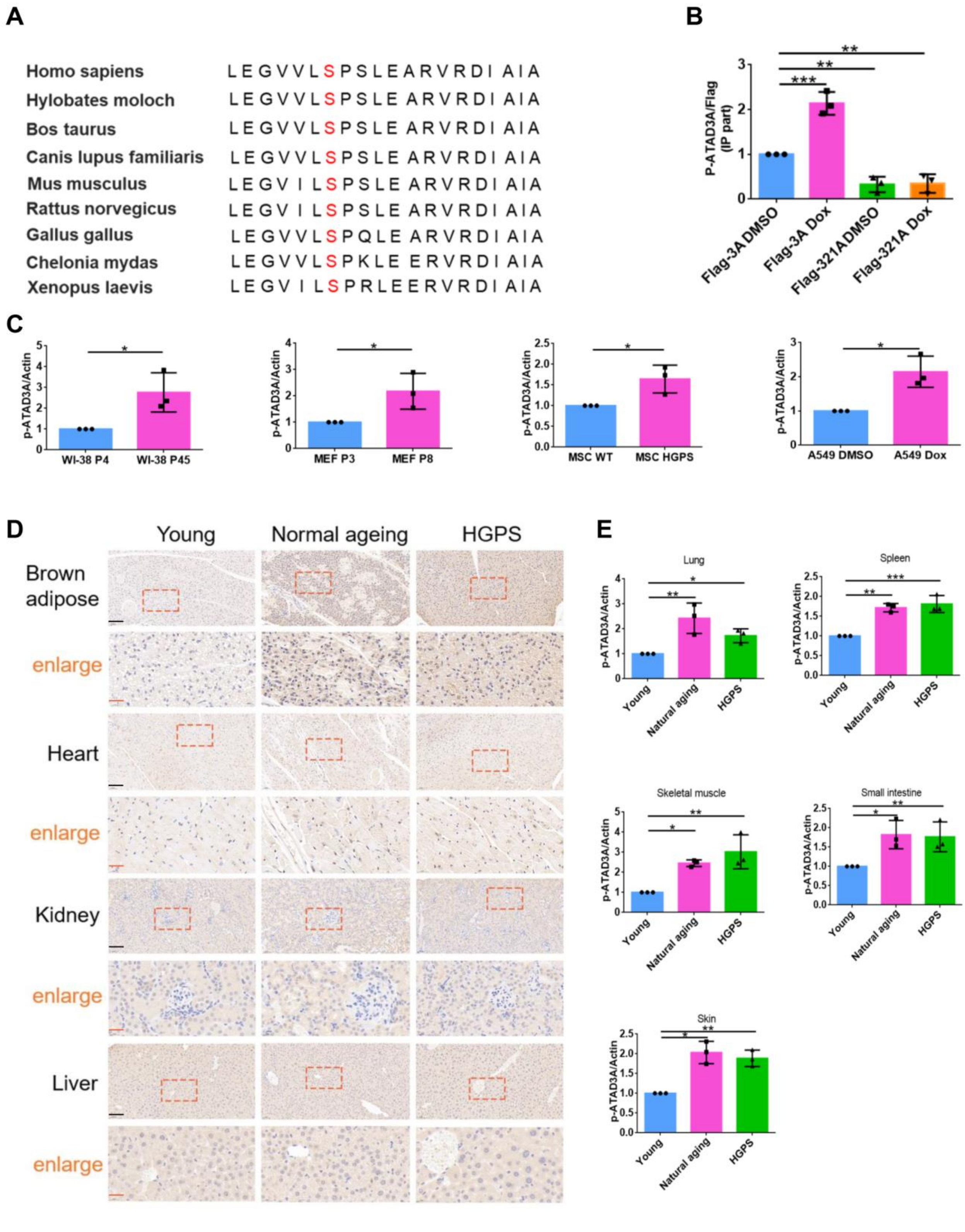

Figure S3. Conserved Ser321 site of ATAD3A and its phosphorylation in cells and mice tissues. (A) Sequence alignment of ATAD3A proteins from the indicated species, showing the conservation of Ser321. (B) Quantification of the immunoblot analysis as presented in Figure 3C (n=3). $^{**}P < 0.01$, $^{***}P < 0.001$ (one-way ANOVA with Dunnet post-hoc test). (C) Quantification of the immunoblot analysis as presented in Figure 3D

(n=3). *P < 0.05 (Unpaired two-tailed T-test). (D) Immunohistochemistry staining of p-ATAD3A positive cells in the brown adipose, heart, kidney, liver from young (3 months), natural aging (20-21 months) and HGPS (6 months) mice (n=3). Areas outlined by squares are enlarged at below. Scale bars (black), 60 μm. Scale bars (orange), 20 μm. (E) Quantification of the immunoblot analysis as presented in Figure 3F (n=3). *P < 0.05, **P < 0.01, ***P < 0.001 (one-way ANOVA with Dunnet post-hoc test). All data are presented as the mean ± SD.

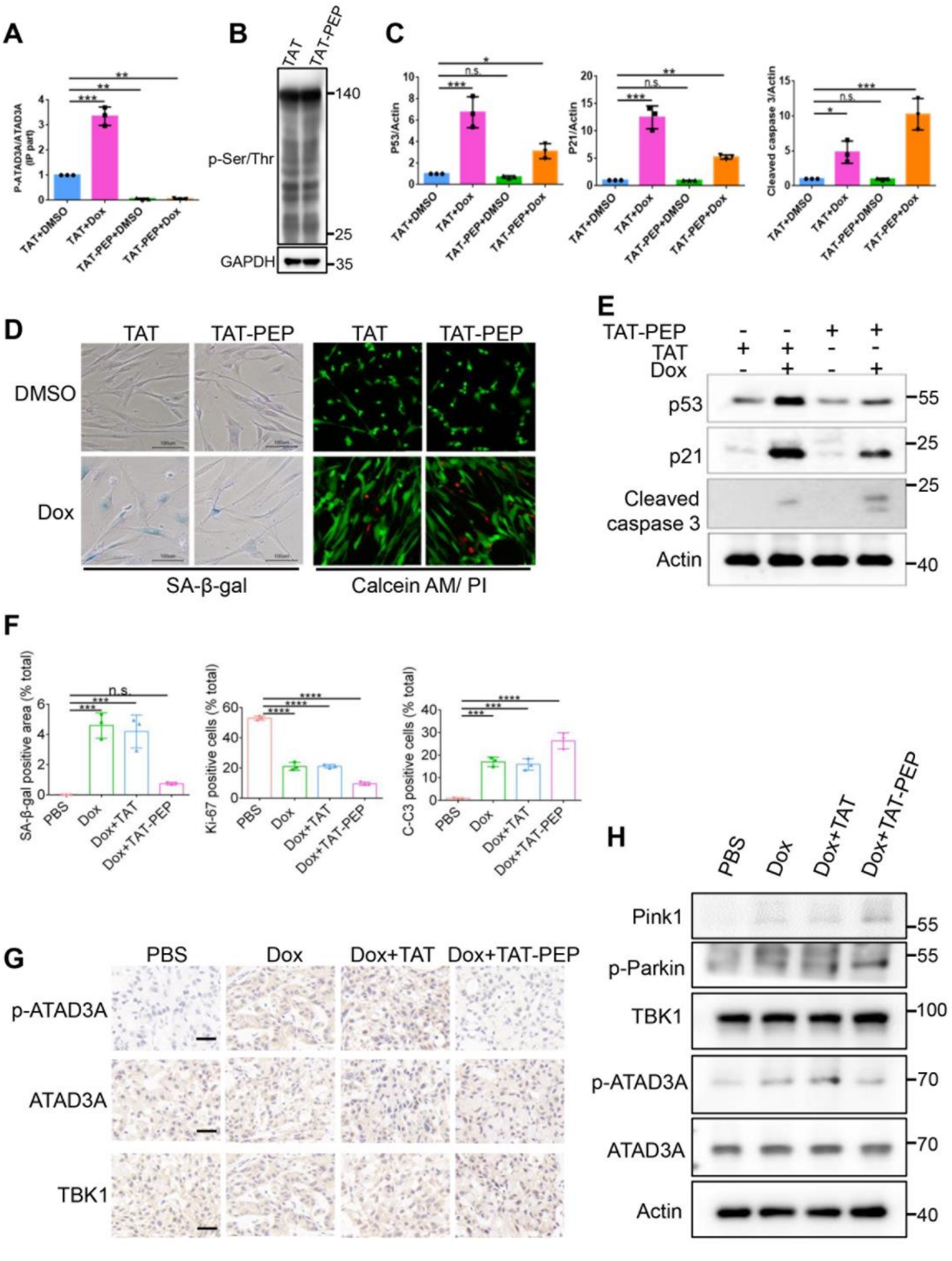

Figure S4. Blocking phosphorylation of ATAD3A increases cell sensitivity to doxorubicin. (A) Quantification of the immunoblot analysis as presented in Figure 4B (n=3). **P < 0.01, ***P < 0.001 (one-way ANOVA with Dunnet post-hoc test). (B) Immunoblot analysis of phosphorylated Ser/Thr in A549 cells treated with either TAT (50 μM) or TAT-PEP (50 μM) for 96 h (n=3). (C) Quantification of the immunoblot analysis as presented in Figure 4D (n=3). n.s., not significant, *P < 0.05, **P < 0.01, ***P < 0.001 (one-way ANOVA with Dunnet post-hoc test). (D) Detection of cellular senescence and cell death in WI-38 cells treated with doxorubicin (150 nM) plus TAT (50 μM) or TAT-PEP (50 μM), using SA-β-gal staining and Calcein AM/PI staining (n=3). Cells were initially induced with DMSO or doxorubicin (150 nM) in combination with TAT (50 μM) or TAT-PEP (50 μM) for 3 days, followed by additional culturing for 3 days. Scale bars, 50 μm. (E)

Immunoblot analysis of cellular senescence marker (p53, p21) and a cell apoptosis marker (cleaved caspase 3) in WI-38 cells treated with doxorubicin (150 nM) plus TAT or TAT-PEP (50 μM) (n=3). Cells were initially induced with DMSO or doxorubicin (150 nM) in combination with TAT (50 μM) or TAT-PEP (50 μM) for 3 days, followed by additional culturing for 3 days. (F) Quantification of β-gal positive area, Ki-67 positive, and cleaved caspase 3 positive cells shown in Figure 4I (n=3). Analysis was based on three different images covering dozens of cells using the ImageJ software. C-C3: Cleaved caspase 3. n.s., not significant, ***$P < 0.001$, ****$P < 0.0001$ (one-way ANOVA with Tukey post-hoc test). (G, H) Immunohistochemistry (IHC) and immunoblot analyses of indicated proteins in mouse tumors (n=3). Scale bars, 100 μm. All data are presented as the mean ± SD.

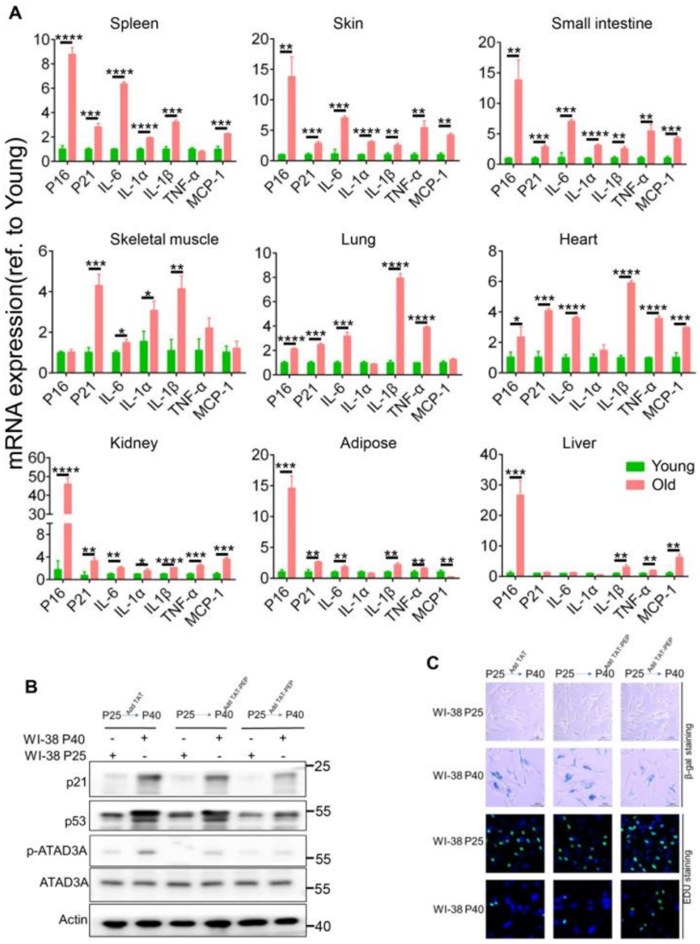

Figure S5. TAT-PEP delays senescence rather than reverses senescence. (A) Expression of p16, p21, IL-6, IL-1α, IL-1β, TNF-α and MCP-1 was analyzed by RT-qPCR in various tissues from young (3 months) and aged mice (20-21 months) (n=3). *P < 0.05, **P < 0.01, ***P < 0.001, ****P < 0.0001 (Unpaired two-tailed T-test). All data are presented as the mean ± SD. (B, C) Immunoblot and β-gal staining indicate cellular senescence markers, while EDU staining shows cellular proliferation (n=3). WI-38 cells at passage 25 were divided into three groups: the first group was continuously treated with TAT until passage 40; the second group was treated with TAT-PEP only at passage 40; and the third group was continuously treated with TAT-PEP until passage 40. Scale bars, 100 μm.

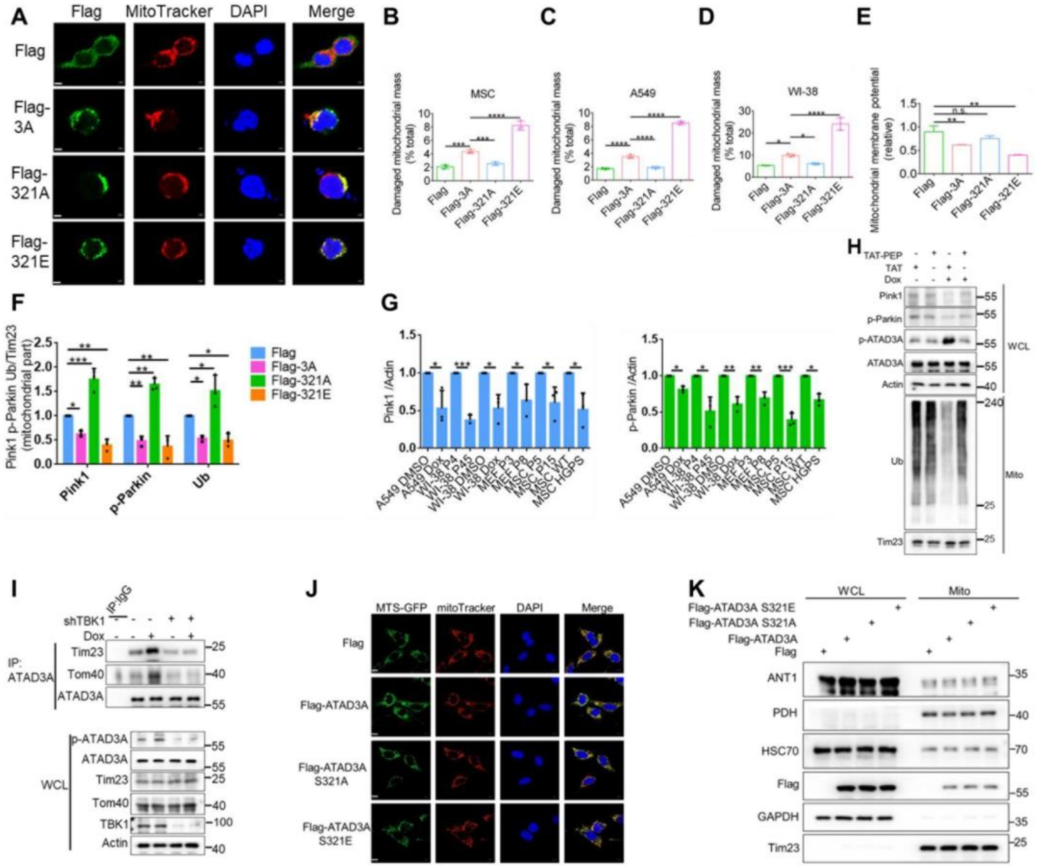

Figure S6. Phosphorylated ATAD3A increases mitochondrial damage. (A) Confocal imaging of ATAD3A, ATAD3A S321A, ATAD3A S321E (green) and MitoTracker (red) localization in 293T cells transfected with Flag-vector, Flag-ATAD3A, Flag-ATAD3A S321A or Flag-ATAD3A S321E (n=3). Scale bars, 5 μm. (B-D) Quantification of damaged mitochondrial mass as presented in Figure 6C (n=3). *P < 0.05, ***P< 0.001, ****P< 0.0001 (one-way ANOVA with Tukey post-hoc test). (E) Mitochondrial membrane potential detection in 293T cells transfected with Flag- vector, Flag-ATAD3A, Flag-ATAD3A S321A or Flag-ATAD3A S321E. Cells were incubated with JC-1 fluorescent probe, and signals were quantified (n=3). n.s., not significant, **P < 0.01 (one-way ANOVA with Tukey post-hoc test). (F) Quantification of the immunoblot analysis as presented in Figure 6E (n=3). *P < 0.05, **P < 0.01, ***P < 0.001 (one-way ANOVA with Dunnet post-hoc test). (G) Quantification of the immunoblot analysis as presented in Figure 6G (n=3). *P < 0.05, **P < 0.01, ***P < 0.001 (Unpaired two-tailed T-test). (H) Immunoblot analysis of Pink1, p-Parkin, Ub in the WCL or mitochondrial fraction of A549 cells treated with doxorubicin (150 nM) plus TAT (50 μM) or TAT-PEP (50 μM) (n=3). (I) Immunoblot analysis of the interaction between the indicated proteins and ATAD3A in TBK1 knockdown A549 cells with or without doxorubicin treatment (n=3). (J, K) Immunofluorescence analysis of GFP-MTS (J) and immunoblot analysis of the mitochondrial localization of proteins such as ANT1/PDH/HSC70 (K) in A549 cells overexpressing vector, wild-type ATAD3A, ATAD3A-Ser321A, or ATAD3A-Ser321E (n=3). ANT1: Adenine Nucleotide Translocator 1; PDH: Pyruvate Dehydrogenase. Scale bars, 10 μm. All data are presented as the mean ± SD.

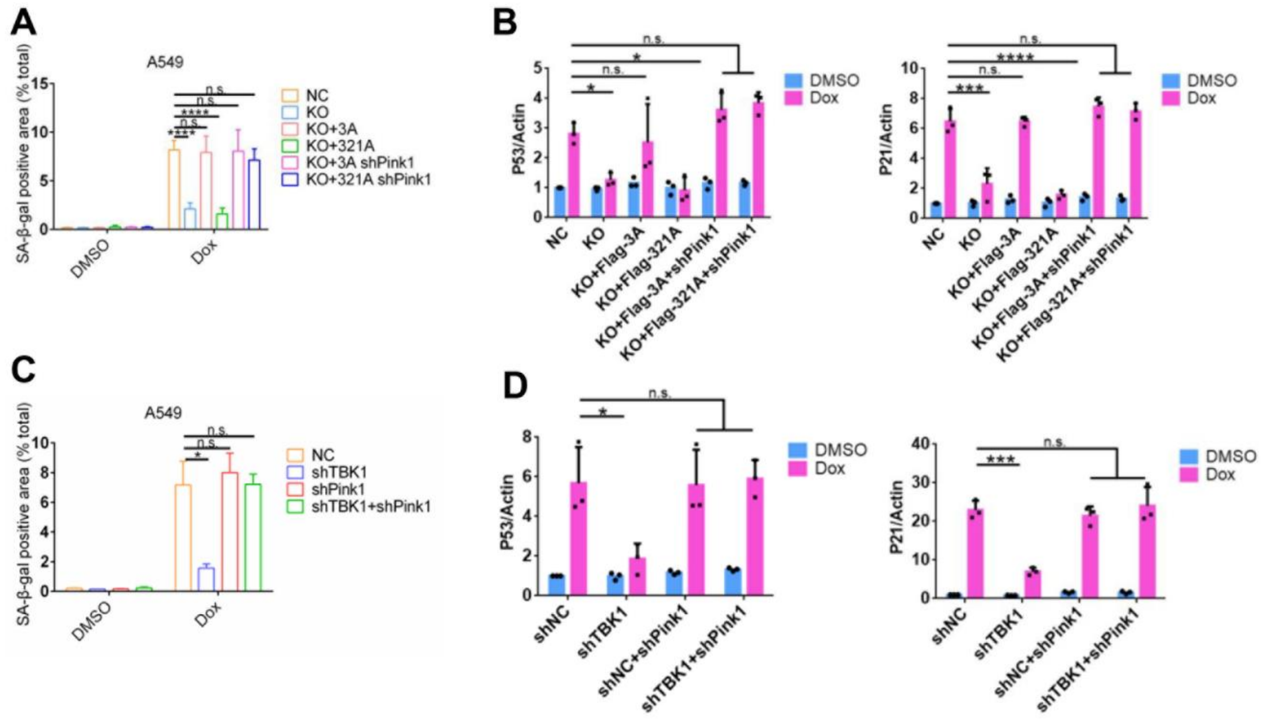

Figure S7. TBK1-ATAD3A axis promotes cellular senescence by inhibiting Pink1 mediated mitophagy. (A) Quantification of SA-β-gal staining as presented in Figure 7A (n=3). Analysis was based on four different images covering dozens of cells using the ImageJ software. n.s., not significant, ****P < 0.0001 (one-way ANOVA with Dunnet post-hoc test). (B) Quantification of the immunoblot analysis as presented in Figure 7B (n=3). n.s., not significant, *P < 0.05, ***P < 0.001, ****P < 0.0001 (one-way ANOVA with Dunnet post-hoc test). (C) Quantification of SA-β-gal staining as presented in Figure 7D (n=3). Analysis was based on four different images covering dozens of cells using the ImageJ software. n.s., not significant, *P < 0.05 (one-way ANOVA with Dunnet post-hoc test). (D) Quantification of the immunoblot analysis as presented in Figure 7E (n=3). n.s., not significant, *P < 0.05, ***P < 0.001 (one-way ANOVA with Dunnet post-hoc test). All data are presented as the mean ± SD.

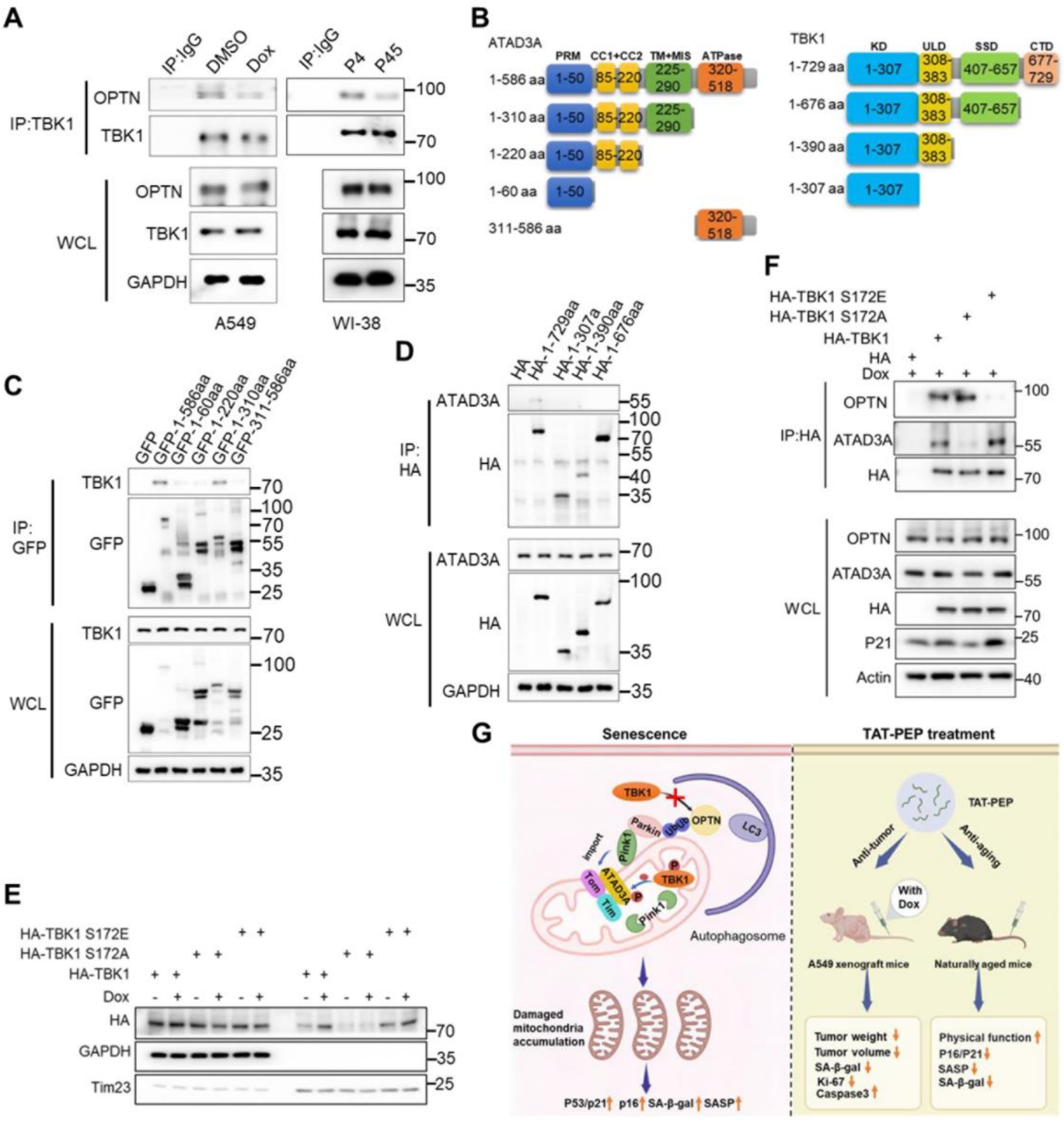

Figure S8. A switch from the TBK1-OPTN-mitophagy activation axis to the TBK1-ATAD3A-Pink1 mitophagy inhibition axis drives senescence. (A) Immunoblot analysis of the interaction between TBK1 and OPTN in normal or senescent A549 and WI-38 cells (n=3). (B) Schematics of ATAD3A and TBK1 deletion mutants. PRM: proline-rich motif, CC1 and CC2: coiled-coil domains, TM+MIS: trans-membrane amphipathic and mitochondrial import signal, KD: kinase domain, ULD: ubiquitin-like domain, SSD: scaffold and dimerization domain, CTD: C-terminal domain. (C) Immunoblot analysis of the interaction between endogenous TBK1 and ATAD3A deletion mutants as described in B (n=3). (D) Immunoblot analysis of the interaction between endogenous ATAD3A and TBK1 deletion mutants as described in B (n=3). (E) Immunoblot analysis of TBK1 mitochondrial localization in doxorubicin-treated A549 cells overexpressing vector, wild-type TBK1, TBK1-Ser172A, or TBK1-Ser172E (n=3). (F) Immunoblot analysis of the interaction between TBK1 and ATAD3A as well as OPTN, in doxorubicin-induced senescent A549 cells overexpressing vector, wild-type TBK1, TBK1-Ser172A, or TBK1-Ser172E (n=3). (G) Schematic representation of the TBK1-ATAD3A-Pink1 senescence-promoting axis and therapies targeting ATAD3A phosphorylation. Left panel, during cellular senescence, TBK1 is activated and localizes to mitochondria, switching TBK1-OPTN interaction to TBK1-ATAD3A interaction. TBK1-mediated phosphorylation of ATAD3A enhances Pink1 mitochondrial import by

promoting the assembly of the mitochondrial membrane complex Tim23-Tom40, thus suppressing mitophagy. This leads to the accumulation of damaged mitochondria and ultimately results in cellular senescence. Right panel, A combination of the ATAD3A phosphorylation blocking peptide TAT-PEP with doxorubicin treatment significantly reduces tumor weight and volume, as well as tumor cell senescence and proliferation, while increasing tumor cell apoptosis. Treating naturally aged mice with TAT-PEP shows improved physical function and a reduction in aging-related signatures, such as the senescence markers p16/p21, SASP, and SA-β-gal activity. All data are presented as the mean ± SD.

Table. S1 List of primers used for generating mutants.

**Gene**	
ATAD3A-S321A	F- GTGTTGTGCTCGCCCCCAGCCTGGA<br>R- TCCAGGCTGGGGGCGAGCACAACAC
ATAD3A-S321E	F- GTGTTGTGCTCGAACCCAGCCTGGA<br>R- TCCAGGCTGGGTTCGAGCACAACAC
ATAD3A-S289A	F- AGTGAGGGAGACGGCCCGCATCACG<br>R- CGTGATGCGGGCCGTCTCCCTCACT

Table. S2 List of primers used for lentivirus construction

**shRNA Primers**	
shTBK1 1#	CCGGGCGGCAGAGTTAGGTGAAATTCTCGAG
shTBK1 3#	CCGGGCAGAACGTAGATTAGCTTATCTCGAG
shPink1	CCGGCGGCTGGAGGAGTATCTGATACTCGA

Table. S3 List of primers used for real time PCR.

**RT-qPCR primers**	
Human ATAD3A	F-TCTTTGTTCTCGGCTCCCAC<br>R-GGTAAACAGACCCCAGCTCC
Human Pink1	F-CCTGGAGTGTGAAACGCTCT<br>R-CTCCCACCCTCACCATTCAC
Human Actin	F-AAGGATTCCTATGTGGGCGAC<br>R-CGTACAGGGATAGCACAGCC
Mouse p16	F- CCCAACGCCCCGAACT<br>R- GCAGAAGAGCTGCTACGTGAA
Mouse p21	F- GTCAGGCTGGTCTGCCTCCG<br>R- CGGTCCCGTGGACAGTGAGCAG
Mouse IL-6	F- CCCCAATTTCCAATGCTCTCC<br>R- CGCACTAGGTTTGCCGAGTA
Mouse IL1-α	F- AGGGAGTCAACTCATTGGCG<br>R- TGGCAGAACTGTAGTCTTCGT
Mouse IL1-β	F- TCCTGTGTGATGAAAGACGGCAC<br>R- GTGCTGATGTACCAGTTGGGGAAC
Mouse TNF-α	F- ATGAGAAGTTCCCAAATGGC<br>R- CTCCACTTGGTGGTTTGCTA
Mouse MCP-1	F- AGCCAACTCTCACTGAAGCC<br>R- GGACCCATTCCTTCTTGGGG
Mouse Actin	F- GATGTATGAAGGCTTTGGTC<br>R- TGTGCACTTTTATTGGTCTC
